# Clinically friendly smart hydrogel boosts cuproptosis and PD-L1 upregulation to enhance anti-tumor immunotherapy

**DOI:** 10.1016/j.mtbio.2025.102268

**Published:** 2025-09-09

**Authors:** Baiyang Fu, Guangyan Li, Yuan Yao, Mingfu Zhang, Yesheng Zhong, Xi Wang, Yichi Chen, Wenlong Liang, Yao Wang, Haiyun Lin, Yutong Zhang, Qiguang Du, Zhongkai Xu, He Cui, Liping Shi, Xi Chen, Jianguo Zhang

**Affiliations:** a Department of Breast Surgery, The Second Affiliated Hospital of Harbin Medical University, No. 246 Xuefu Road, Harbin, 150000, China; b Department of Ultrasound, The Second Affiliated Hospital of Harbin Medical University, No. 246 Xuefu Road, Harbin, 150000, China; c National Key Laboratory of Science and Technology on Advanced Composites in Special Environments, Harbin Institute of Technology, No. 92 Xidazhi Street, Harbin, 150000, China; d Department of Ultrasound, Harbin Medical University Cancer Hospital, No. 150 Haping Road, Harbin, 150000, China

**Keywords:** TNBC, Cancer immunotherapy, Cuproptosis, Hydrogel, PD-L1

## Abstract

Triple-negative breast cancer (TNBC) faces the challenge of limited treatment efficacy due to its highly invasive ability and its immunosuppressive microenvironment. This study found that cuproptosis, as an emerging therapeutic strategy, has unique therapeutic potential in TNBC. Nevertheless, the delivery difficulties of cuproptosis-inducing agents and the limited efficacy of single drugs restrict the clinical application of cuproptosis therapy. Herein, a temperature/pH dual-responsive composite hydrogel was developed to load Elesclomol-Cu (ES-Cu) and glucose oxidase (GOx) (ES-Cu&GOx@FFC). ES and Cu^2+^ can synergistically trigger cuproptosis in TNBC, and GOx can not only inhibit tumor metabolism by mediating glucose deprivation but also initiate the Fenton reaction by continuously generating H_2_O_2_ and synergizing with copper ions, driving a potent chemodynamic therapy (CDT). Furthermore, ES-Cu&GOx@FFC could significantly improve the immunosuppressive landscape and upregulate programmed death-ligand 1 (PD-L1) expression in TNBC. Combined therapy experiments showed that the combination treatment of ES-Cu&GOx@FFC and αPD-L1 achieved more than 90 % tumor volume regression. In summary, this study provides new insights into the therapeutic role of cuproptosis in TNBC, and integrates cuproptosis, starvation therapy, CDT, and immunotherapy through smart responsive hydrogels, providing an innovative solution for the treatment of TNBC.

## Graphical abstract

**Figure:**
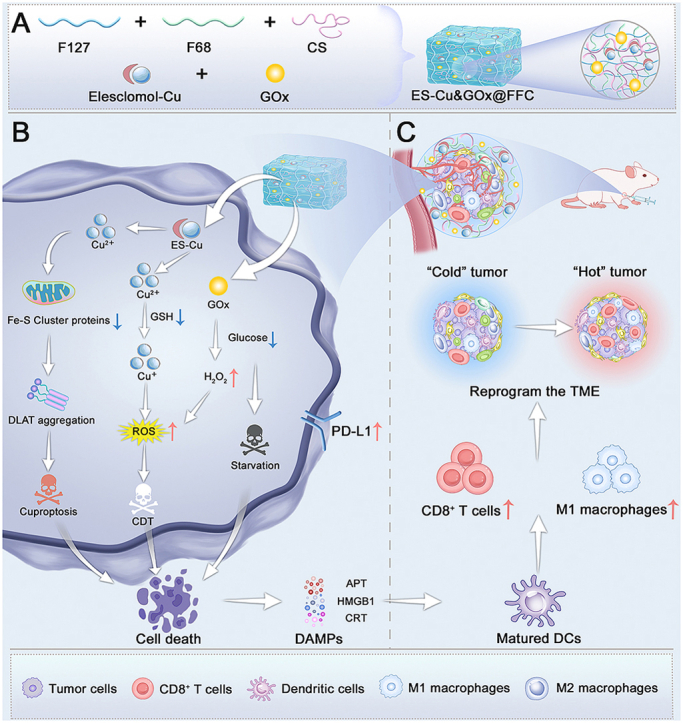

## 1 Introduction

Triple-negative breast cancer (TNBC) is the subtype of breast cancer with the most limited treatment options due to the lack of clear therapeutic targets [1]. Although immune checkpoint blockade (ICB) therapy represented by programmed death ligand 1 (PD-L1) inhibitors has brought clinical benefits to some patients, the overall response rate is less than 20 % and secondary drug resistance is prone to occur [2]. The reasons for the limited efficacy of ICB therapy can be attributed to two aspects: first, TNBC is a typical "cold tumor" and its immunosuppressive tumor microenvironment (TME) weakens the anti-tumor activity of T lymphocytes [3]; second, the expression level of PD-L1 on the surface of TNBC tumor cells is highly heterogeneous, which means that ICB treatment must strictly rely on biomarkers to screen the applicable population, greatly limiting its clinical universality [4]. In addition, although traditional chemotherapy and radiotherapy can transiently activate anti-tumor immunity by inducing immunogenic cell death (ICD), their nonspecific killing effects cause non-negligible short-term and long-term toxicities such as bone marrow suppression and organ damage, which seriously hinder their application [5]. Therefore, to break through the bottleneck of TNBC treatment, it is necessary to develop new combined strategies that can overcome the immunosuppression of TME and synergistically enhance ICB response.

Cuproptosis is a copper-dependent cell death mechanism discovered in recent years. Elesclomol (ES) is a highly efficient copper ion (Cu^2+^) carrier that specifically chelates Cu^2+^ to form an ES-Cu complex. ES, with its unique mitochondrial targeting, promotes the transport of copper from the extracellular space into the mitochondria. Subsequently, copper ions bind to acylated DLATs, triggering protein oligomerization and downregulation of Fe-S cluster proteins, which together lead to cytotoxic stress and ultimately induce cuproptosis [6]. On the other hand, due to the abnormal metabolism of tumor cells, which show significant glucose addiction, cutting off the glucose supply has become a key strategy for metabolic intervention therapy [7]. Glucose oxidase (GOx) is a natural enzyme catalyst that can effectively catalyze the conversion of glucose into gluconic acid and simultaneously generate hydrogen peroxide (H_2_O_2_), achieving tumor starvation therapy by consuming local glucose [8]. In addition, chemodynamic therapy (CDT) is based on the Fenton or Fenton-like reaction of metal ions, which converts low-toxic H_2_O_2_ into highly cytotoxic reactive oxygen species (ROS) [9], such as hydroxyl radicals (⋅OH), which can effectively kill tumor cells [10]. However, the antioxidant barrier formed by insufficient endogenous H_2_O_2_ concentration and excessive glutathione (GSH) levels in the tumor microenvironment seriously weakens the efficacy of CDT [11]. In this context, the combined application of ES-Cu and GOx may solve this problem: the H_2_O_2_ catalyzed and generated by GOx can make up for the lack of H_2_O_2_ in the TME, while Cu^2+^ can consume excess GSH and convert it into oxidized glutathione (GSSG). Finally, copper ions can convert H_2_O_2_ into highly toxic ·OH through a Fenton-like reaction, which can break through the antioxidant defense barrier and significantly enhance the tumor-killing efficiency of ROS [12]. Meanwhile, cuproptosis and CDT can not only serve as a cell killing mechanism, but also induce immunogenic cell death (ICD) as a tool for immune regulation. Cuproptosis and CDT can promote antigen presentation by inducing cells to release damage-associated molecular patterns (DAMPs) such as high-mobility histone 1 (HMGB1) and ATP, laying the foundation for reversing the immunosuppressive TME [13]. This multi-mechanism synergistic cell killing strategy can avoid the secondary drug resistance induced by single-mechanism anti-tumor strategies by blocking the activation of compensatory pathways. More importantly, recent studies have shown that copper accumulation and glucose deprivation within the tumor can synergistically upregulate PD-L1 expression on the tumor surface [12], providing a basis for resolving the heterogeneity of PD-L1 expression in TNBC.

Although the combined application of ES-Cu and GOx has promising therapeutic prospects, the lack of targeting specificity of ES-Cu and GOx, coupled with their rapid clearance and metabolism in the blood, leads to limited accumulation in tumor sites and increases the risk of damage to normal tissues, thus limiting their clinical application [14]. Therefore, developing an intelligent delivery system that can respond to TME-specific signals and achieve controlled release of ES-Cu and GOx is the key to improving efficacy and safety. Recently, many bio-nanomaterials and medical hydrogels have been developed for drug loading. Although bio-nanomaterials have active or passive tumor-targeting effects in systemic administration, recent studies have shown that only a very small portion of nanoparticles (0.7 %) can reach tumor tissues under intravenous administration, and their nonspecific accumulation may induce toxicity risks to important organs [15]. In contrast, injectable anti-tumor hydrogels, due to their unique three-dimensional network characteristics and physicochemical properties, can achieve precise drug delivery through local peritumoral injection, which can reduce systemic exposure [16]. Medical drug-loaded hydrogels can not only serve as in situ "drug warehouses" to achieve sustained drug release but can also degrade spontaneously, maximizing tumor-killing effects while minimizing systemic toxic side effects [17]. It is particularly noteworthy that, targeting the key characteristics of the TNBC microenvironment (physiological temperature of 37 °C, acidic environment of pH 6.5–6.8 [18]), a temperature/pH dual-responsive smart hydrogel drug delivery platform was designed, which is more practical in anti-tumor applications.

Chitosan (CS) is a natural polymer widely found in nature. CS hydrogel has good pH responsiveness and biocompatibility. However, the poor solubility and lack of temperature responsiveness of traditional CS hydrogels limit their practical applications [19]. Therefore, researchers have introduced thermosensitive components to construct a temperature/pH dual-responsive system to overcome the above limitations, among which Poloxamers are an ideal thermosensitive candidate material [20]. Poloxamers (such as Pluronic F127 and Pluronic F68) are amphiphilic triblock copolymers composed of polyoxyethylene-polyoxypropylene-polyoxyethylene (PEO–PPO–PEO) and are a common thermosensitive nonionic surfactant. Poloxamers polymer molecules self-assemble into a structure with water-insoluble PPO as the core and water-soluble PEO as the shell so that drugs with poor water solubility can be encapsulated in the hydrophobic PPO core [21]. Nevertheless, single poloxamer-based hydrogels have inherent defects such as poor stability, low mechanical strength, and rapid dissolution in biological environments, which make it difficult to meet the requirements of long-term sustained release of drugs [22]. In the current study, we combined CS with Pluronic F127/F68 to develop a ternary composite hydrogel (FFC), which is characterized by stable co-loading of hydrophobic/hydrophilic drugs while circumventing the limitations of CS and poloxamers. What is particularly important is that all components of FFC have clear clinical translation safety. F127/F68 are FDA-approved pharmaceutical excipients, and CS is an FDA-approved food additive, which avoids the potential toxicity risks of industrially produced synthetic nanomaterials (such as metal-organic frameworks, polymer nanoparticles, etc.).

**Scheme 1:**
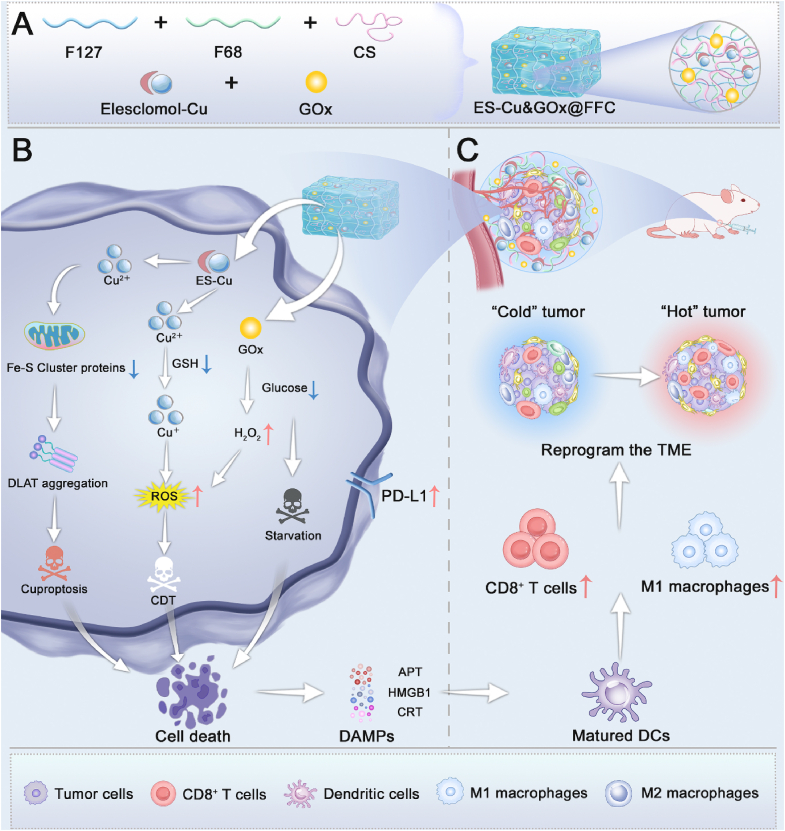
**Schematic diagram of ES-Cu&GOx@FFC hydrogel for TNBC treatment.** (A) Schematic diagram of ES-Cu&GOx@FFC hydrogel preparation. (B) Biological mechanism of ES-Cu&GOx@FFC hydrogel inducing TNBC cell cuproptosis, CDT, starvation, upregulation of PD-L1 expression, and ICD. (C) ES-Cu&GOx@FFC hydrogel reverses the immunosuppressive tumor microenvironment, which enhances TNBC immunotherapy.

In this study, we revealed for the first time the unique potential of cuproptosis in the treatment of TNBC from the perspective of drug sensitivity and immune activation. Inspired by these findings, we constructed a temperature/pH dual-responsive composite hydrogel loaded with ES-Cu and GOx (ES-Cu&GOx@FFC). ES-Cu&GOx@FFC has thermosensitive gelation (gelation at 37 °C), pH responsiveness (acidic TME triggers drug release), and injectability. The hydrogel can precisely target the tumor site and respond to microenvironmental signals to achieve intelligent drug delivery, providing an ideal platform for local treatment of TNBC. The released ES-Cu induces cuproptosis via mitochondrial copper accumulation, while GOx-mediated glucose deprivation not only directly inhibited tumor proliferation but also triggered CDT via the generation of H_2_O_2_. In addition, cuproptosis and CDT can synergistically trigger ICD, further promote dendritic cells (DCs) maturation, and CD8^+^ T cell infiltration, and reverse the M2 polarization phenotype of tumor-associated macrophages. This reprogramming of the immune microenvironment can cooperate with cancer starvation and cuproptosis-induced upregulation of tumor surface PD-L1 expression to effectively enhance the αPD-L1 therapy of TNBC. In summary, ES-Cu&GOx@FFC provides an innovative solution for local treatment and immune activation of TNBC (Scheme 1).

## 2 Results and discussion

### 2.1 Cuproptosis has therapeutic potential in TNBC

**Fig. 1:**
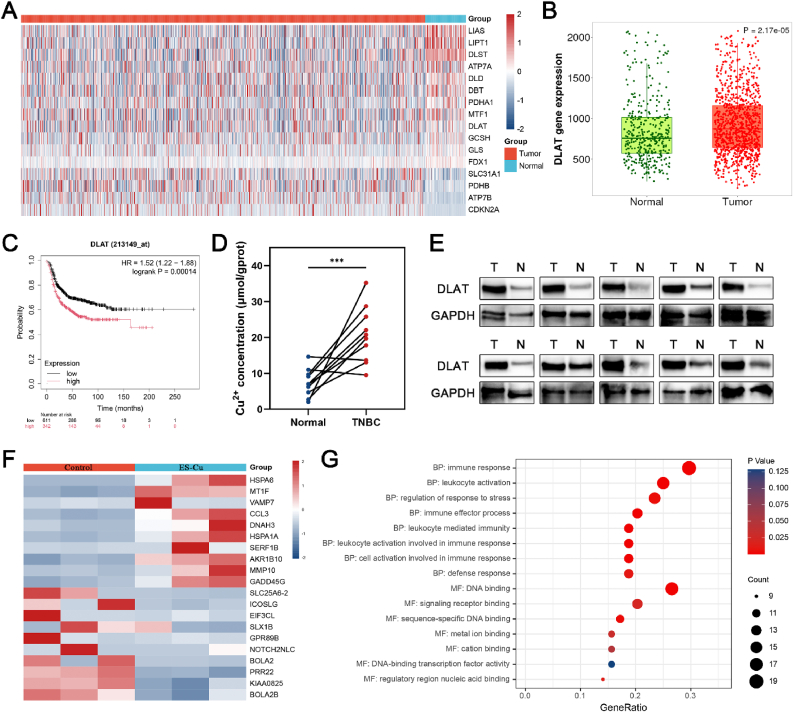
**The therapeutic potential of cuproptosis in TNBC.** (A) Heat map depicting the expression levels of cuproptosis-related genes in breast cancer and adjacent normal tissues. (B) Differential expression of DLAT mRNA in breast cancer and adjacent normal tissues. (C) Kaplan–Meier curves of DFS in patients with high or low expression of DLAT in TNBC. (D) Paired line scatter plots showing copper concentrations in TNBC (n = 10) and adjacent normal tissues (n = 10). (E) Western blot was used to detect the expression of DLAT in breast cancer tissues and adjacent normal tissues of 10 TNBC patients. (F) Heat map depicting the transcriptome changes after ES-Cu induced cuproptosis in TNBC. (G) GO enrichment analysis of differentially expressed genes after ES-Cu induced cuproptosis in TNBC.

To evaluate the therapeutic potential of cuproptosis in breast cancer, we systematically analyzed the mRNA expression levels of 16 cuproptosis-related genes in breast cancer and adjacent tissue samples based on the TCGA database [23,24]. Heat map analysis showed that breast cancer tissues and adjacent normal samples showed significant differential expression in the transcriptional levels of cuproptosis-related genes (Fig. 1A). Among them, DLAT, FDX1, and LIAS are the key regulatory genes of cuproptosis. Compared with the adjacent normal breast tissue, the mRNA expression level of DLAT in breast cancer samples was significantly increased (Fig. 1B). Based on Kaplan-Meier survival curve analysis, the 5-year disease-free survival rate of patients with high DLAT expression was significantly lower than that of patients with low DLAT expression (Fig. 1C). In addition, the correlation analysis between DLAT and FDX1 and LIAS further revealed that DLAT expression was positively correlated with both FDX1 and LIAS expression (Fig. S1A and B). These bioinformatics results showed that high expression of DLAT was not only associated with the malignant phenotype and poor prognosis of TNBC but also closely related to the expression of cuproptosis core regulatory genes FDX1 and LIAS. To further verify the results of bioinformatics analysis, we analyzed the copper concentration and DLAT protein expression in 10 pairs of TNBC primary lesions and paired adjacent clinical tissue specimens. The results showed that the copper level in TNBC tissues was significantly increased (Fig. 1D), and the DLAT protein expression was also higher than that in adjacent tissue specimens (Fig. 1E). In summary, TNBC showed significant abnormal copper metabolism and DLAT overexpression characteristics, suggesting that it is sensitive to the treatment of cuproptosis.

In addition, we further explored the relationship between cuproptosis and the tumor immune microenvironment. Correlation analysis showed that DLAT was positively correlated with PD-L1 expression in TNBC tissues (Fig. S2A). Tumor-infiltrating immune cells were analyzed using the TIMER algorithm, and the DLAT expression level was positively correlated with the infiltration abundance of CD4^+^ T cells, CD8^+^ T cells, and DCs (Fig. S2B–D). To understand its molecular mechanism, we performed RNA-Seq analysis on ES-Cu-treated MDA-MB-231 cells. Heat map results showed that the upregulation of MT1F (which plays an important role in maintaining metal homeostasis and defending against heavy metal toxicity [25]), AKR1B10 (which plays a role in anti-oxidative stress [26]), and HSPA1A (which resists stress and protects cells from damage [27]) in MDA-MB-231 cells reflects the cellular compensatory defense against copper overload to maintain copper homeostasis. This cellular stress response confirms ES-Cu-induced cuproptosis (Fig. 1F). Next, we performed Gene Ontology (GO) enrichment analysis on the differentially expressed genes after inducing cuproptosis in TNBC. The results showed that the differentially expressed genes were mainly enriched in immune-related biological processes such as "immune response" and "leukocyte activation" (Fig. 1G). We further summarized the expression of genes related to "immune response" and "leukocyte activation" and presented them in the form of a heat map (Fig. S3A). Correlation analysis results showed that 80.9 % (17/21) of the genes related to "immune response" and "leukocyte activation" were correlated with PD-L1 expression, CD4^+^ T cells, CD8^+^ T cells, and DCs infiltration abundance in breast cancer (Fig. S3B). These evidences indicate that inducing cuproptosis not only has the potential to directly kill TNBC but also can enhance PD-L1 expression, promote immune cell infiltration, and reprogram the immune microenvironment, providing a theoretical basis for the synergistic effect of cuproptosis therapy and immunotherapy.

### 2.2 Preparation and characterization of ES-Cu&GOx@FFC hydrogel

**Fig. 2:**
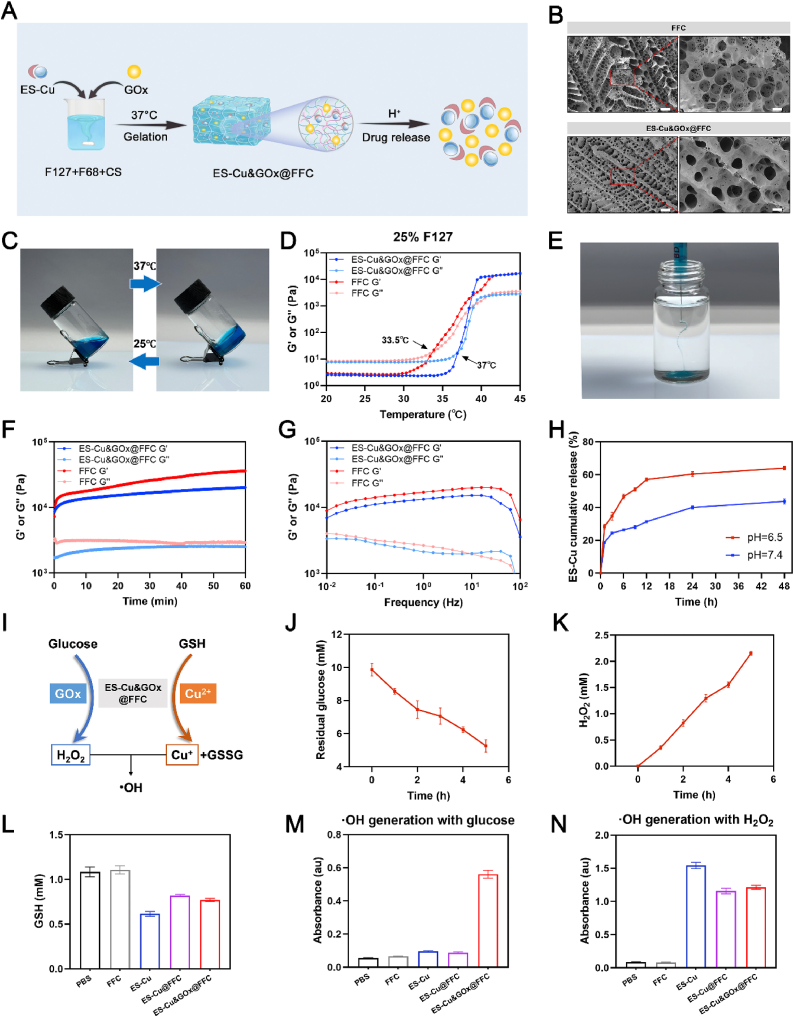
**Preparation and characterization of ES-Cu&GOx@FFC hydrogels.** (A) Schematic diagram of the preparation and pH-responsive release of ES-Cu&GOx@FFC hydrogels. (B) SEM images of freeze-dried FFC hydrogels and ES-Cu&GOx@FFC hydrogels (Scale bar [left] = 10 μm, Scale bar [right] = 2 μm). (C) Photographs of the gel-sol state transition of ES-Cu&GOx@FFC hydrogels at 25 °C and 37 °C. (D) Evolution of G′ and G″ of FFC and ES-Cu&GOx@FFC hydrogels in the range of 20–45 °C. (E) Photographs of ES-Cu&GOx@FFC hydrogels injected into PBS. (F) Time evolution of G′ and G″ of FFC and ES-Cu&GOx@FFC hydrogels at 37 °C. (G) Frequency evolution of G′ and G″ of FFC and ES-Cu&GOx@FFC hydrogels at 37 °C. (H) Cumulative release of ES-Cu from ES-Cu&GOx@FFC gels in buffer solutions of different pH. (I) Schematic diagram of the cascade catalytic reaction induced by ES-Cu&GOx@FFC. (J) Residual glucose content after reaction with ES-Cu&GOx@FFC at different time points. (K) H_2_O_2_ generation from the reaction between ES-Cu&GOx@FFC and glucose at different time points. (L) GSH content after incubation of different treatment groups. (M) ·OH generation of different treatment groups in the presence of glucose only. (N) ·OH generation of different treatment groups in the presence of H_2_O_2_ only. The indicated results represent the mean ± SD of three independent experiments.

To fully realize the potential of cuproptosis in the treatment of TNBC, this study selected the cuproptosis inducer ES-Cu as the core drug and integrated GOx to build a multi-mechanism synergistic treatment system. Aiming at the characteristics of the TNBC microenvironment, we designed and developed a ternary composite hydrogel based on Pluronic F127/Pluronic F68/CS, aiming to achieve controlled drug release and maintain effective drug concentration in the tumor. As shown in Fig. 2A, CS was dissolved in glacial acetic acid, magnetically stirred until completely dissolved, and then F127 and F68 were added in sequence. After mixing, they were allowed to stand at 4 °C for 12 h to allow them to swell fully and dissolve completely. Finally, ES-Cu and GOx were uniformly mixed with the FFC solution to form an ES-Cu&GOx@FFC injectable hydrogel. Most current nanomedicines have diverse compositions and complex structures. The cumbersome synthesis pathways often lead to reduced reproducibility of results. In addition, the controllable and large-scale production of nanomedicines also faces significant challenges [28]. The ES-Cu&GOx@FFC designed in this study can significantly enhance its clinical translation potential due to its advantages such as low cost, simple synthesis process and good reproducibility. To develop an injectable hydrogel with physiological temperature responsiveness, we optimized its thermosensitive properties by adjusting the concentration of Pluronic F127. The experiment found that the effective concentration range of F127 in FFC hydrogel is 25 %–30 %. When the concentration of F127 is lower than 25 %, the system cannot form a stable gel network at 37 °C, and when the concentration exceeds 30 %, F127 will be difficult to dissolve. Therefore, we selected three F127 concentration gradients, including 30 %, 27.5 %, and 25 %, for further temperature sensitivity optimization studies. Temperature sweep rheological tests showed that with increasing temperature, the storage modulus (G′) of all concentration systems showed an “S-shaped” growth and eventually exceeded the loss modulus (G″). The temperature at which G″ = G′ represents the lower critical solution temperature (LCST). We found that the F127 concentration was negatively correlated with the LCST. The LCSTs of the three concentrations of FFC hydrogels were 26 °C, 29.5 °C, and 33.5 °C, respectively (Fig. S4). The FFC hydrogel carrier with an F127 concentration of 25 % showed the most suitable LCST (33.5 °C) for in-situ gelation in the body. Finally, we used this ratio to synthesize FFC hydrogels and then loaded ES-Cu and GOx for further experiments.

As shown in the scanning electron microscope in Fig. 2B, the interior of the FFC hydrogel presents a typical three-dimensional porous network structure, while the loading of ES-Cu and GOx does not significantly change its micromorphology. Excellent thermosensitivity and injectability are prerequisites for drug-loaded hydrogels to be suitable for in situ tumor treatment. ES-Cu&GOx@FFC can achieve a rapid sol-gel transition (t < 3 min) at a physiological temperature of 37 °C (Fig. 2C). We also evaluated the thermal transition of ES-Cu&GOx@FFC gel by rheological test (Fig. 2D). At low temperature or room temperature, G′ of ES-Cu&GOx@FFC is less than G″, indicating that ES-Cu&GOx@FFC is in a sol state at this temperature. When the temperature rises to 37 °C, G′ and G″ begin to rise rapidly, and the growth rate of G′ is greater than G″. Finally, G’ is greater than G″, confirming the rapid formation of a three-dimensional network structure and showing its excellent thermal sensitivity. To evaluate clinical operability, we used a 29G (0.33 mm) insulin syringe for simulated injection and found that ES-Cu&GOx@FFC hydrogel had good injectability (Fig. 2E). Next, we used the time scan (0–60min) and frequency scan (0.01–100Hz) modes of the rheometer to test the FFC hydrogel and ES-Cu&GOx@FFC hydrogel samples at 37 °C. Fig. 2F, G shows that the hydrogel system always maintains G′>G″ within the measurement range, and the modulus of the FFC hydrogel does not change significantly after ES-Cu and GOx are loaded. The above rheological evidence jointly confirms that the thermosensitive hydrogel can maintain excellent mechanical stability during the physiological temperature phase transition and drug loading. Due to the abnormal metabolism of tumors, the pH in the tumor microenvironment is acidic. Therefore, using this property to design a drug delivery platform can not only improve the anti-tumor efficacy of drugs but also reduce systemic toxicity while enhancing the anti-tumor efficacy. Given the acidic microenvironment of TNBC, we evaluated the pH-responsive release behavior of the ES-Cu&GOx@FFC hydrogel drug delivery system. As shown in Fig. 2H, the drug-loaded gel was placed in buffer solutions of pH = 6.5 (simulating the microenvironment of TNBC) and pH = 7.4 (simulating the physiological environment) for in vitro release experiments. The results showed that the cumulative release of ES-Cu in 24 h under pH = 6.5 conditions reached 60.3 %, which was significantly higher than 39 % under pH = 7.4 conditions. Similarly, the cumulative release of GOx under acidic conditions was higher than that under neutral conditions (Fig. S5). These results indicate that ES-Cu&GOx@FFC gel exhibits good pH-responsive release behavior, and this pH-dependent release can be attributed to the protonation of chitosan amino groups under acidic conditions, which leads to increased intramolecular electrostatic repulsion and increased swelling [29].

Fig. 2I shows the cascade catalytic reactions of ES-Cu&GOx@FFC, including the consumption of GSH mediated by copper ions, the catalytic effect of glucose in the presence of GOx, and the continuous generation of ·OH from Cu^+^ and H_2_O_2_ through a Fenton-like reaction. To verify the catalytic activity of GOx in ES-Cu&GOx@FFC, glucose was incubated with the drug-loading system. As the incubation time increased, the residual glucose content decreased sharply, while the generated H_2_O_2_ increased rapidly within 4 h, which indicated the high catalytic efficiency of the drug-loading system (Fig. 2J and K). In addition, after incubation of GSH solution with different experimental groups, the GSH content of the ES-Cu&GOx@FFC group was significantly decreased, and the GSSG content was significantly increased (Fig. 2L, Fig. S6). Given the role of Cu^2+^ in the consumption of GSH, we further displayed the Cu concentration in the supernatant of different groups (Fig. S7). Based on the above results, we speculate that the consumption of GSH and the generation of GSSG may be caused by the redox effect of Cu^+^/Cu^2+^ electron pairs [12]. Next, we used a hydroxyl radical content detection kit to evaluate the ·OH generation ability of ES-Cu&GOx@FFC. In the presence of ·OH, thiobarbituric acid (TBA) can be condensed into a detectable colored product, and the generation of ·OH is evaluated by measuring the absorbance of the product. In the presence of exogenous glucose alone, ES-Cu&GOx@FFC showed significantly higher absorbance values than other groups at 120 min (Fig. 2M). In the presence of exogenous H_2_O_2_ alone, the ES-Cu group had a higher absorbance value than the ES-Cu@FFC group and ES-Cu&GOx@FFC, which was due to the reduced ·OH generation rate caused by the drug sustained release effect of the hydrogel as a carrier (Fig. 2N).

In summary, this study successfully constructed an injectable smart hydrogel with dual temperature/pH responsiveness. This drug delivery platform uses a combination of clinically friendly materials to load hydrophobic cuproptosis inducers, and the loaded Cu^2+^ and GOx also show excellent catalytic properties. These results show that ES-Cu&GOx@FFC has the potential to kill triple-negative breast cancer cells in a multi-mechanism synergistic manner (cuproptosis, starvation therapy, CDT).

### 2.3 In vitro antitumor ability and cuproptosis activation of ES-Cu&GOx@FFC hydrogel

**Fig. 3:**
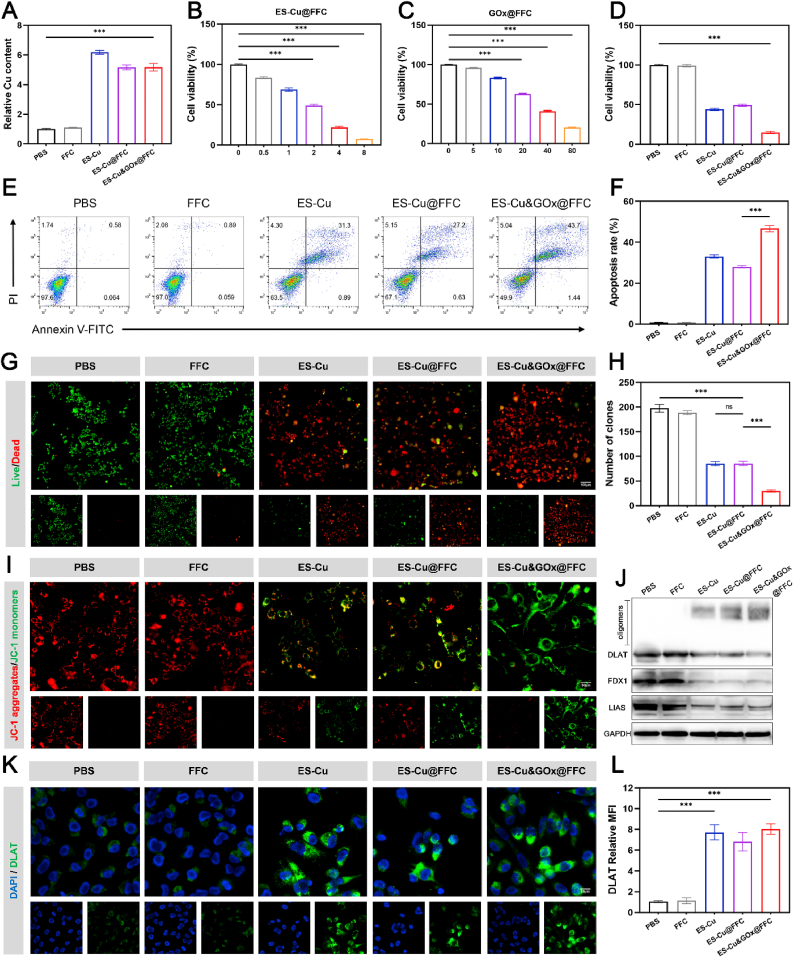
**In vitro antitumor and cuproptosis induction of 4T1 by ES-Cu&GOx@FFC hydrogels.** (A) Intracellular uptake of copper by 4T1 cells in different treatment groups. (B) Cell viability of 4T1 cells after 24 h of treatment with different concentrations of ES-Cu@FFC. (C) Cell viability of 4T1 cells after 24 h of treatment with different concentrations of GOx@FFC. (D) Cell viability of 4T1 cells after 24 h of treatment with different treatment groups. (E, F) Flow cytometry analysis of Annexin V-FITC/PI double staining to determine apoptotic cell populations (Q2+Q3). (G) Fluorescence images of live (green)/dead (red) staining of 4T1 cells after different treatments (Scale bar = 100 μm). (H) Quantitative analysis of colony numbers after different treatments. (I) Fluorescence images of JC-1 aggregates (red) and JC-1 monomers (green) in 4T1 cells after different treatments (Scale bar = 50 μm). (J) Western blot of DLAT, DLAT oligomers, FDX1, and LIAS protein expressions in 4T1 cells after different treatments. (K, L) Representative immunofluorescence images and quantitative analysis of DLAT (green) after indicated treatment (Scale bar = 50 μm). The indicated results represent the mean ± SD of three independent experiments. ns, not significant, ∗P < 0.05, ∗∗P < 0.01, ∗∗∗P < 0.001. (For interpretation of the references to color in this figure legend, the reader is referred to the Web version of this article.)

Given that the function of ES as a copper ion carrier is to mediate the specific transmembrane transport of copper, and the level of intracellular copper accumulation is positively correlated with cytotoxicity, we treated 4T1 cells with different experimental groups and used a kit to detect the intracellular copper content, to quantitatively evaluate the copper delivery efficiency of ES-Cu&GOx@FFC. The results showed that the intracellular copper concentrations of the ES-Cu, ES-Cu@FFC, and ES-Cu&GOx@FFC groups were 6.2, 5.1, and 5.2 times that of the PBS control group, respectively. Although the ES-Cu&GOx@FFC group was slightly lower than the free ES-Cu group due to the hydrogel sustained release effect, it was still significantly higher than the control group (Fig. 3A). In short, ES-Cu&GOx@FFC can significantly promote the transport of copper into tumor cells and effectively increase the copper content in 4T1 cells.

Subsequently, we used the CCK-8 assay to detect the in vitro antitumor activity of ES-Cu&GOx@FFC and the synergistic antitumor effect of ES-Cu and GOx. The results showed that the cytotoxicity of ES-Cu@FFC increased significantly with the increase of ES concentration in the hydrogel (Fig. 3B). Similarly, a decrease in cell viability caused by increased GOx content in GOx@FFC hydrogel was also observed (Fig. 3C). Next, we used different experimental groups to treat 4T1 cells. ES-Cu&GOx@FFC hydrogel (ES concentration was 2 μM, GOx concentration was 20 μg/mL) could reduce the cell survival rate to 14.8 %, which was stronger than the two single-drug hydrogels of ES-Cu@FFC with a drug concentration of 2 μM (survival rate was 49.2 %) and GOx@FFC with a drug concentration of 20 μg/mL (survival rate was 62.9 %), indicating the synergistic effect of ES-Cu and GOx (Fig. 3D). In particular, the survival rate of FFC-treated 4T1 cells did not change compared with that of the PBS-treated control group, indicating that the FFC ternary composite hydrogel has excellent biocompatibility. Observation under a light microscope revealed that 4T1 cells co-incubated with ES-Cu showed obvious damage to the integrity of the cell membrane. In addition, the number of cells with damaged cell membranes in the ES-Cu&GOx@FFC treatment group was the largest (Fig. S8). This morphological change is similar to the characteristic morphology of cuproptosis observed in previous studies [6,30]. Annexin V-FITC/PI double staining flow cytometry analysis showed that the total apoptosis rate of 4T1 cells induced by ES-Cu&GOx@FFC was 46.7 %, which was 1.4 times higher than that of cells treated with ES-Cu (33 %) and 1.7 times higher than that of cells treated with ES-Cu@FFC (27.9 %) (Fig. 3E and F). We further stained the cells with live/dead dyes to detect live and dead cells. Consistent with the above results, in the ES-Cu&GOx@FFC group, most cells were stained red, indicating that the ES-Cu&GOx@FFC hydrogel group had the highest ability to kill cancer cells (Fig. 3G). We also verified the anti-tumor effect of ES-Cu&GOx@FFC by plate colony formation experiments. The results showed that the number of colonies in the ES-Cu&GOx@FFC group was about 84 % lower than that in the PBS group (Figure S9, Fig. 3H). To further explore the mechanism of cell death, we detected mitochondrial membrane potential and protein expression of key regulatory genes of cuproptosis. The core mechanism of cuproptosis involves oxidative stress and metabolic disorders caused by copper ion overload in mitochondria. The mitochondrial membrane potential (MMP) status can be assessed using the JC-1 fluorescent probe, which emits red fluorescence in healthy mitochondria with high MMP and green fluorescence in mitochondria with low MMP. This dynamic change can directly reflect the degree of damage to mitochondrial function during cuproptosis [31]. Fluorescence microscopy observations showed that 4T1 cells treated with ES-Cu&GOx@FFC exhibited the strongest green fluorescence and the weakest red fluorescence, in contrast to the bright red fluorescence characteristics of healthy mitochondria in the PBS group, indicating that mitochondrial damage was the most severe in the ES-Cu&GOx@FFC group (Fig. 3I). In addition, the molecular mechanism of cuproptosis involves the excessive accumulation of copper ions in mitochondria that can directly bind to DLAT, leading to DLAT oligomerization, followed by proteotoxic stress and cellular cuproptosis. When cuproptosis occurs, in addition to DLAT oligomerization, the consumption of Fe-S cluster proteins FDX1 and LIAS is also a key indicator of cuproptosis [32]. After 24 h of ES-Cu&GOx@FFC treatment, the WB results of 4T1 cells showed an increase in DLAT oligomerization and a decrease in the expression levels of FDX1 and LIAS, which are consistent with the hallmark molecular events of cuproptosis (Fig. 3J, Fig. S10). In addition, we also visualized DLAT oligomerization by immunofluorescence imaging. As shown in Fig. 3K, cells treated with PBS or FFC had only negligible DLAT accumulation, while obvious aggregation of DLAT was observed in tumor cells treated with ES-Cu, ES-Cu@FFC, and ES-Cu&GOx@FFC. Statistical analysis found that the fluorescence intensity of the ES-Cu and ES-Cu&GOx@FFC groups was higher than other groups (Fig. 3L). In general, the ES-Cu&GOx@FFC hydrogel system can achieve efficient killing of TNBC cells in vitro through the cuproptosis mechanism.

### 2.4 ES-Cu&GOx@FFC hydrogel mediated CDT, ICD, and upregulation of PD-L1 expression

**Fig. 4:**
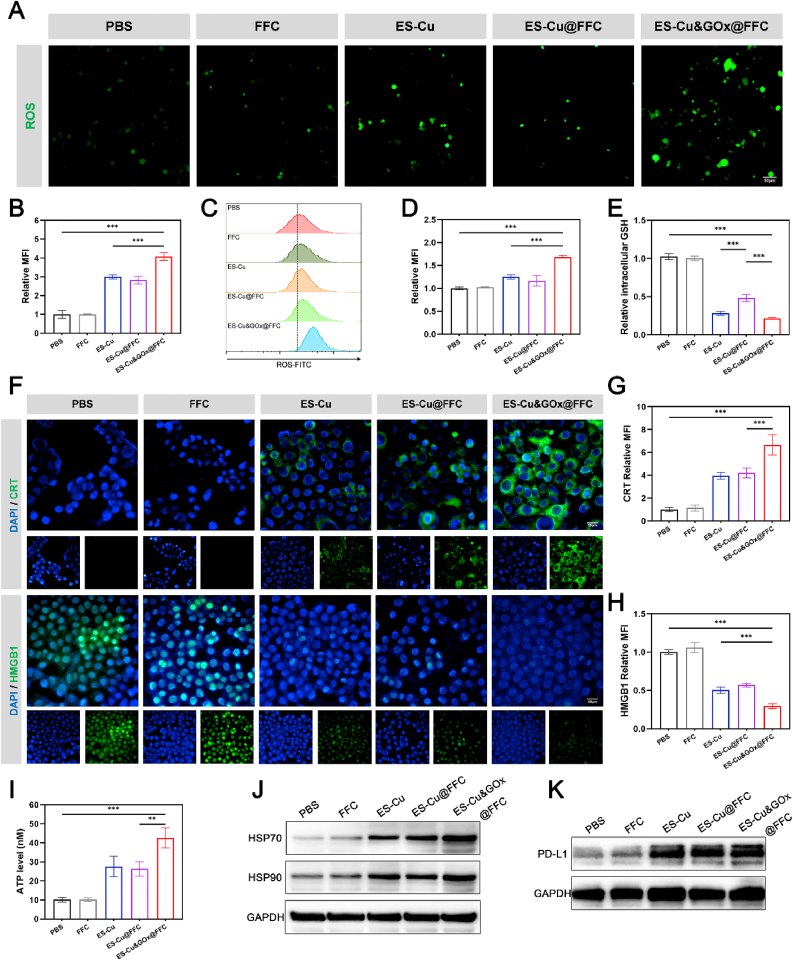
**ES-Cu&GOx@FFC hydrogel-mediated CDT, ICD, and PD-L1 upregulation in vitro.** (A, B) Representative fluorescence images and mean fluorescence intensity of intracellular ROS in different treatment groups detected by DCFH-DA (green) probe (Scale bar = 50 μm). (C, D) Representative flow cytometry histograms and quantitative analysis of intracellular ROS in different treatment groups analyzed by flow cytometry. (E) Relative GSH levels in 4T1 cells after treatment with different treatment groups. (F–H) Representative immunofluorescence images and quantitative analysis of CRT (green) and HMGB1 (green) after indicated treatment (Scale bar = 50 μm). (I) ATP levels in the culture supernatant of 4T1 cells after incubation with indicated treatments for 24 h. (J) WB analysis of HSP70 and HSP90 expression levels in 4T1 cells in different treatment groups. (K) WB analysis of PD-L1 expression levels in 4T1 cells in different treatment groups. The indicated results represent the mean ± SD of three independent experiments. ns, not significant, ∗P < 0.05, ∗∗P < 0.01, ∗∗∗P < 0.001. (For interpretation of the references to color in this figure legend, the reader is referred to the Web version of this article.)

We verified the occurrence of CDT by detecting the generation of ROS and the degree of GSH consumption in cells. To demonstrate the ability of ES-Cu&GOx@FFC hydrogel to generate ROS, cells were stained with 2′,7′-dichlorofluorescein diacetate (DCFH-DA) fluorescent probe and observed by fluorescence microscopy or measured by flow cytometry for the fluorescence intensity of different treatment groups. Fig. 4A, B shows that the fluorescence intensity of the ES-Cu and ES-Cu@FFC groups was significantly increased compared with the control group. This increase in ROS levels is attributed to the ROS produced by the Fenton-like reaction mediated by Cu ions. In addition, the ES-Cu&GOx@FFC treatment group has the highest fluorescence intensity, because the excess H_2_O_2_ generated by the GOx-catalyzed reaction provides a continuous substrate for the Fenton-like reaction, forming an amplification effect of ROS generation. Flow cytometry detection showed the same conclusion as fluorescence microscopy (Fig. 4C and D). The reduction of intracellular GSH has the potential to sensitize ROS-based treatments and promote cuproptosis [33]. We measured intracellular GSH levels using a GSH content assay kit. In the presence of GSH, DTNB can be reduced to a detectable colored product, TNB, and the intracellular GSH level can be assessed by measuring the absorbance of this product. As shown in Fig. 4E, after treatment with ES-Cu, ES-Cu@FFC, and ES-Cu&GOx@FFC, the intracellular glutathione levels decreased by 72.6 %, 53.0 %, and 79.0 %, respectively. This reduction can be attributed to the oxidative nature of Cu^2+^ released from ES-Cu&GOx@FFC [34]. These results indicate that ES-Cu&GOx@FFC hydrogels can effectively induce CDT of TNBC in vitro.

Recent studies have shown that cuproptosis and CDT can induce ICD to enhance antitumor immune responses [30,35]. ICD is characterized by the release of a series of DAMPs, including calreticulin (CRT) exposed on the cell membrane surface, secreted ATP, and HMGB1 released by dying cells. These released DAMPs can act as an “eat me” signal, be recognized by DCs, induce DCs maturation, promote T cell activation, and ultimately trigger a strong anti-tumor immune response to eradicate tumor cells [32]. Specifically, surface exposure of CRT can interact with the CD91 receptor on DCs, enabling them to effectively phagocytose dying cells. Furthermore, the N-terminal fragment of CRT can activate the PI3K/Akt pathway in DCs, inducing DCs maturation [36]. In addition to acting as a "find me" signal, extracellular ATP can also mediate immunostimulatory effects by activating the NLRP3 inflammasome and subsequently secreting IL-1β, which is crucial for DC-mediated immunogenic death [37]. HMGB1, by binding to TLR4 on DCs, mediates proinflammatory effects and effectively cross-presents tumor antigens [38]. In addition, the upregulated expression of HSP70 and HSP90 is also one of the hallmarks of ICD. Studies have shown that increased expression of HSP70 and HSP90 in tumor cells is associated with DCs activation, and HSP expression can enhance tumor cell immunogenicity and improve tumor clearance in vivo [39]. As shown in Fig. 4F and G, we used immunofluorescence staining to detect the migration of CRT to the cell membrane. The results showed that the green fluorescence with the strongest fluorescence intensity was observed in cells treated with ES-Cu&GOx@FFC, proving the massive transfer of CRT. Next, we also observed the secretion of HMGB1 from the nucleus to the extracellular space. The results showed that the green fluorescence and DAPI overlapped in 4T1 cells treated with PBS and FFC, while only negligible HMGB1 fluorescence was observed in the cells of the ES-Cu&GOx@FFC treatment group, which proved the release of HMGB1 (Fig. 4F–H). In addition, we also quantitatively analyzed the ATP released by 4T1 cells, and the results showed that the highest relative ATP concentration was detected in the supernatant of cells treated with ES-Cu&GOx@FFC, which was 4.2 and 1.6 times that of the PBS group and ES-Cu@FFC group, respectively (Fig. 4I). At the same time, the WB results in Fig. 4J and Fig. S11 showed that ES-Cu&GOx@FFC significantly increased the expression levels of HSP70 and HSP90 proteins in 4T1 cells. These results indicate that ES-Cu&GOx@FFC effectively enhances the immunogenicity of tumor cells by inducing ICD in tumor cells. The study also found that cuproptosis and glucose starvation therapy have a synergistic effect on the upregulation of PD-L1 in tumor cells [12], so we further studied the effects of different treatment groups on PD-L1 expression at the cellular level. Western blot results showed that ES-Cu&GOx@FFC treatment can significantly increase PD-L1 expression on the surface of 4T1 (Fig. 4K, Fig. S12).

In summary, the above findings confirm the hypothesis that ES-Cu&GOx@FFC can induce cuproptosis and CDT, and further induce ICD, which can increase the immunogenicity of tumor cells and activate anti-tumor immune responses. Moreover, ES-Cu&GOx@FFC can also upregulate PD-L1, suggesting that ES-Cu&GOx@FFC combined with αPD-L1 may be a promising form of cancer treatment.

### 2.5 RNA-seq analysis of 4T1 treated with different treatment groups

**Fig. 5:**
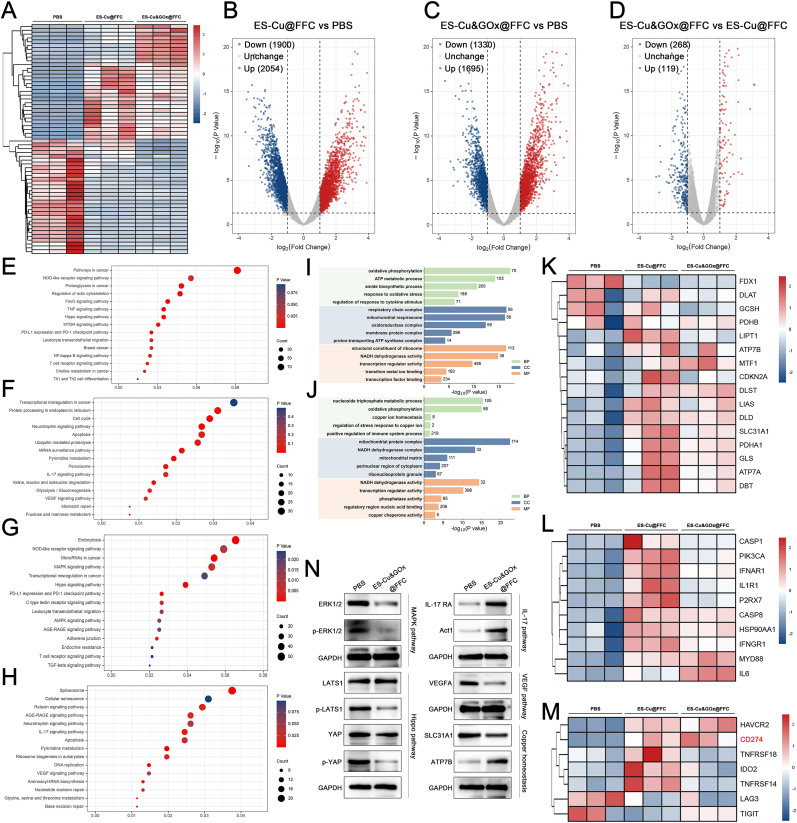
**RNA-Seq of 4T1 cells treated with various treatments.** (A) Heatmap of DEGs expression of cells treated with PBS, ES-Cu@FFC, and ES-Cu&GOx@FFC. (B–D) Volcano plots showing the DEGs between different treatment groups. (E) KEGG enrichment analysis of differentially upregulated genes between cells treated with PBS and ES-Cu@FFC. (F) KEGG enrichment analysis of differentially downregulated genes between cells treated with PBS and ES-Cu@FFC. (G) KEGG enrichment analysis of differentially upregulated genes between cells treated with PBS and ES-Cu&GOx@FFC. (H) KEGG enrichment analysis of differentially downregulated genes between cells treated with PBS and ES-Cu&GOx@FFC. (I) GO enrichment analysis of differentially expressed genes between cells treated with PBS and ES-Cu@FFC. (J) GO enrichment analysis of differentially expressed genes between cells treated with PBS and ES-Cu&GOx@FFC. (K) Heatmap of cuproptosis-related gene expression in cells of the indicated treatment groups. (L) Heatmap of ICD-related gene expression in cells of the indicated treatment groups. (M) Heatmap of immune checkpoint-related gene expression in cells of the indicated treatment groups. (N) Expression of immune-related signaling pathways and copper homeostasis-related proteins in 4T1 cells.

To understand the anti-tumor mechanism of ES-Cu@FFC and ES-Cu&GOx@FFC hydrogels, we performed RNA-seq on 4T1 cells treated with PBS or ES-Cu@FFC or ES-Cu&GOx@FFC for 24 h. The heat map clearly showed the changes in transcript levels in different treatment groups compared with the control group (Fig. 5A). We screened DEGs using a strict threshold (|Log_2_FC|≥1, p < 0.05) and visualized DEGs using a volcano plot. Compared with the control group, ES-Cu@FFC resulted in 2054 upregulated genes and 1900 downregulated genes (Fig. 5B). ES-Cu&GOx@FFC treatment resulted in 1695 upregulated genes and 1330 downregulated genes (Fig. 5C). In addition, compared with cells treated with ES-Cu@FFC, cells treated with ES-Cu&GOx@FFC had 119 upregulated genes and 268 downregulated genes, suggesting that GOx in the drug delivery system had an independent contribution to the effect on DEGs (Fig. 5D).

Kyoto encyclopedia of genes and genomes (KEGG) enrichment analysis showed that the upregulated DEGs after ES-Cu@FFC treatment were mainly enriched in pathways such as "TNF signaling pathway" and "PD-L1 expression and PD-1 checkpoint pathway" (Fig. 5E). The downregulated DEGs after ES-Cu@FFC treatment were mainly enriched in pathways such as "IL-17 signaling pathway" and "Glycolysis/Gluconeogenesis" (Fig. 5F). This indicates that ES-Cu@FFC treatment can affect the energy metabolism, inflammatory response, and immune activation of 4T1. In addition, the up-regulated DEGs after ES-Cu&GOx@FFC treatment were mainly enriched in pathways such as "PD-L1 expression and PD-1 checkpoint pathway" and "Leukocyte transendothelial migration" (Fig. 5G). The down-regulated DEGs after ES-Cu&GOx@FFC treatment were mainly enriched in pathways such as "Apoptosis" and "DNA replication" (Fig. 5H). This indicates that ES-Cu&GOx@FFC treatment can not only activate the immunogenicity of 4T1 cells but also break the dynamic balance between tumor proliferation and death. Based on the results of KEGG enrichment analysis, our drug-loaded hydrogel can not only kill tumors, but also suggests its ability to activate immunity. To verify the above conclusion, we detected the immune-related pathways enriched in differentially expressed genes after ES-Cu&GOx@FFC treatment. MAPK is a pathway that is abnormally activated in malignant tumors. Inhibiting the activity of this pathway can sensitize TNBC to immunotherapy [40]. WB results showed that the expression of ERK1/2 and phosphorylated ERK1/2 proteins was downregulated, indicating that ES-Cu&GOx@FFC inhibited the MAPK pathway (Fig. 5N, Fig. S13A). Abnormal activation of the Hippo signaling pathway is closely related to the immune escape of TNBC [41]. The addition of ES-Cu&GOx@FFC can inhibit p-LATS1 and p-YAP in 4T1 cells and significantly reduce the ratios of p-LATS1/LATS1 and p-YAP/YAP, which means that ES-Cu&GOx@FFC can negatively regulate the Hippo signaling pathway (Fig. 5N, Fig. S13B and C). Studies have shown that activation of the IL-17 signaling pathway in tumor cells can upregulate PD-L1 expression and increase CD8^+^ T cell infiltration [42]. By measuring the expression levels of IL-17 RA and Act1, we found that ES-Cu&GOx@FFC activated the IL-17 signaling pathway (Fig. 5N, Fig. S13D). Finally, we examined VEGF expression. VEGF is not only closely associated with tumor angiogenesis, but its inhibitors, combined with PD-1/PD-L1, have shown significant clinical value in the treatment of advanced TNBC [43]. By detecting the VEGF expression levels in cells and supernatant, it was found that ES-Cu&GOx@FFC can inhibit VEGF (Fig. 5N, Fig. S13E and F). These experimental results demonstrate that ES-Cu&GOx@FFC hydrogel can influence immune-related pathways in tumor cells to achieve immune activation and demonstrate potential for immunotherapy sensitization.

GO enrichment analysis found that "response to oxidative stress" and "regulation of response to cytokine stimulus" were enriched in the ES-Cu@FFC treatment group (Fig. 5I). The DEGs in the ES-Cu&GOx@FFC treatment group were mainly enriched in "copper ion homeostasis", "regulation of stress response copper ion", and "positive regulation of immune system process" (Fig. 5J). The results of GO enrichment analysis showed that the drug-loaded hydrogel designed by us kills tumor cells through three main mechanisms: regulation of copper ion homeostasis, oxidative stress killing, and immune enhancement. We further detected the expression levels of copper homeostasis-related proteins by Western blotting. The results showed that after ES-Cu&GOx@FFC treatment, SLC31A1 expression was downregulated (copper uptake was reduced) and ATP7B expression was upregulated (copper efflux was increased), indicating that the copper homeostasis of the cells was disrupted by ES-Cu&GOx@FFC (Fig. 5N, Fig. S14).

Heat map analysis of the core gene sets of cuproptosis, ICD [44,45], and immune checkpoints [44] showed that ES-Cu@FFC or ES-Cu&GOx@FFC treatment led to transcriptome changes in almost all core genes, especially FDX1, DLAT, and CD274 (PD-L1), whose mRNA expression changes were highly consistent with the protein level changes in previous results, confirming the consistency of regulation at the transcription-translation level (Fig. 5K–M). We used a Venn diagram to display the number of differentially expressed genes in the PBS, ES-Cu@FFC, and ES-Cu&GOx@FFC groups. By intersecting the genes, we identified 52 genes shared by the different treatment groups (Fig. S15A). Next, we constructed a PPI network to further screen the hub genes (Fig. S15B). The results showed that there were five genes with a node degree greater than 10 (IL6, FOS, PTGS2, TLR3, and TLR4). Interestingly, all five genes play important roles in tumor immunotherapy (Fig. S15C).

Based on the KEGG and GO enrichment analysis of DRGs and DEGs of RNA-seq, we verified the synergistic anti-tumor mechanism of ES-Cu&GOx@FFC hydrogel by triggering cuproptosis, CDT, and ICD at the gene expression level. In addition, ES-Cu&GOx@FFC hydrogel can also upregulate the expression of PD-L1, and these conclusions are highly consistent with the results of previous phenotypic experiments.

### 2.6 In vivo antitumor effect of ES-Cu&GOx@FFC hydrogel

**Fig. 6:**
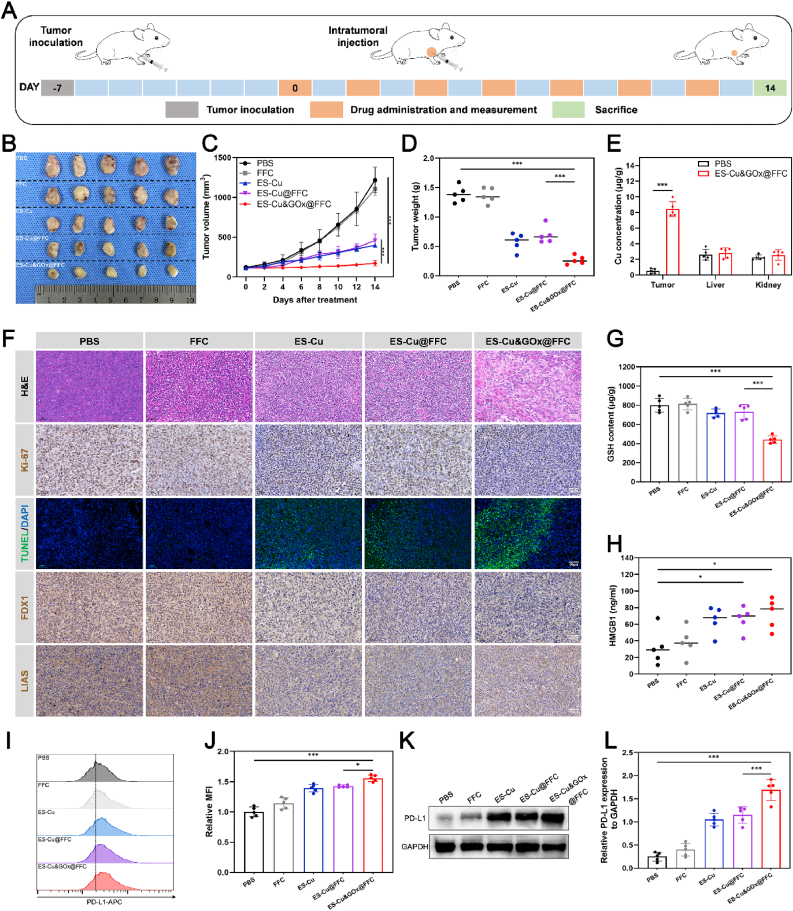
**In vivo antitumor effect of ES-Cu&GOx@FFC hydrogel.** (A) Schematic diagram of the treatment scheme of 4T1 tumor-bearing mice. (B) Photos of dissected tumors on the 14th day after different treatments. (C) Curves of subcutaneous tumor volume change during treatment. (D) Statistical analysis of in vitro tumor weights on the 14th day after different treatments. (E) Cu concentrations in tumors and important organs. (F) H&E, Ki-67, TUNEL, FDX1, and LIAS staining of dissected tumor tissues on the 14th day after different treatments (Scale bar = 50 μm). (G) GSH content in dissected tumors on the 14th day after different treatments. (H) HMGB1 levels in mouse serum on the 14th day after different treatments. (I, J) Flow cytometry was used to detect the expression levels of PD-L1 on the surface of tumor cells in different treatment groups, and the mean fluorescence intensity was quantitatively analyzed. (K, L) The expression levels of PD-L1 in tumor tissues of different treatment groups were detected by WB, and PD-L1 was quantitatively analyzed after normalization with GAPDH. The indicated results represent the mean ± SD of five independent experiments. ns, not significant, ∗P < 0.05, ∗∗P < 0.01, ∗∗∗P < 0.001.

Based on the synergistic antitumor effect verified by in vitro experiments, we further evaluated the in vivo efficacy of ES-Cu&GOx@FFC hydrogel. A BALB/c breast cancer model was established by subcutaneous inoculation of 4T1 cells under the axilla to evaluate its therapeutic effect. On the 7th day after inoculation (referred to as day 0), the tumor-bearing mice were randomly divided into 5 groups (n = 5). As shown in Fig. 6A, different drugs were injected intratumorally every two days starting from day 0 (7 injections in total, with a total dose of 6 mg ES/kg and 149.3 mg GOx/kg). From the day of administration, the tumor volume and body weight of the mice were recorded every other day.

In vivo efficacy evaluation showed that the ES-Cu&GOx@FFC group exhibited the most significant tumor growth inhibition effect. At the treatment endpoint (day 14), the average tumor volume of mice treated with ES-Cu&GOx@FFC was 171.8 mm^3^, which was only 14.1 % of that in the PBS-treated group (1217.9 mm^3^) and 37.5 % of that in the ES-Cu@FFC monotherapy group (457.7 mm^3^) (Fig. 6B, C, Fig. S16). Tumor weight analysis further confirmed the anti-cancer trend of the drug-loaded hydrogel. The mice were sacrificed, and the subcutaneous tumors of the mice were collected and weighed. It was found that the average tumor weight of the mice treated with ES-Cu&GOx@FFC was only 0.27 g, which was 80.9 % lower than that of the PBS-treated group (1.39 g) and 61.6 % lower than that of the ES-Cu@FFC treated group (0.69 g) (Fig. 6D). In addition, we performed H&E, Ki-67 (proliferation marker), and TUNEL (apoptosis/necrosis detection) staining analysis on tumor tissues at the treatment endpoints of different treatment groups to confirm the anti-tumor effect of ES-Cu&GOx@FFC in mice (Fig. 6F). H&E staining found that the ES-Cu&GOx@FFC treatment group showed significant pathological changes compared with other treatment groups, including more extensive nuclear fragmentation and nuclear dissolution. At the same time, ES-Cu&GOx@FFC also showed an inhibitory effect on Ki-67. In addition, most tumor cells in the ES-Cu&GOx@FFC treatment group underwent apoptosis or necrosis, which was confirmed by a high percentage of TUNEL-positive cells (green fluorescence). The above results confirmed its anti-tumor effect in vivo.

To confirm the occurrence of intratumoral cuproptosis in vivo experiments, we first detected the copper concentration in the tumor and major organs (Fig. 6E). In the tumor site, the copper concentration of the ES-Cu&GOx@FFC treatment group was significantly higher than that of the PBS control group. The higher copper content gave it a higher cuproptosis potential. For the liver and kidney, the copper content of the ES-Cu&GOx@FFC group was only slightly higher than that of the model group, indicating that it is safe. Further analysis of cuproptosis markers by immunohistochemistry revealed the downregulation of two Fe-S cluster proteins, FDX1 and LIAS, in the ES-Cu&GOx@FFC treatment group (Fig. 6F). The changes in intratumoral copper accumulation and core regulatory proteins of cuproptosis confirmed that ES-Cu&GOx@FFC directly activated the cuproptosis pathway in mouse tumors. To verify the CDT activity of the drug delivery system, we quantitatively analyzed the GSH level in tumor tissues. In the ES-Cu&GOx@FFC treatment group, GSH decreased significantly, much lower than that of the PBS control group and the ES-Cu@FFC group (Fig. 6G). This GSH depletion indicates that the CDT effect is continuously activated in the tumor microenvironment. Since HMGB1 is the main DAMP released after tumor ICD, we further measured the concentration of HMGB1 in serum. Enzyme-linked immunosorbent assay (ELISA) confirmed that the group treated with ES-Cu&GOx@FFC released a large amount of HMGB1 into the extracellular space (Fig. 6H). The large release of HMGB1 confirmed the ICD effect induced by cuproptosis and CDT, which was highly consistent with the previous in vitro phenotype results. Next, we used flow cytometry to examine PD-L1 expression on the tumor cell surface. The ES-Cu&GOx@FFC-treated group exhibited the highest mean fluorescence intensity (Fig. 6I and J), confirming that ES-Cu&GOx@FFC can upregulate PD-L1 on the tumor surface in vivo. We also reached the same conclusion by using Western blotting to examine PD-L1 protein expression in mouse tumor tissue (Fig. 6K and L).

Although there is still a long way to go for advanced anti-tumor materials from the laboratory to clinical application, the potential clinical value of some key biomarker changes in the in vivo experimental data of ES-Cu&GOx@FFC is encouraging. First, ES-Cu&GOx@FFC hydrogel can significantly reduce the tumor burden in vivo, which means lower recurrence and metastasis rate and longer survival [46]. Furthermore, Ki-67 has independent prognostic value in TNBC. High Ki-67 expression has been associated with increased mortality in TNBC, and persistently elevated Ki-67 expression after treatment indicates high tumor aggressiveness and a high risk of drug resistance [47]. Our experimental results showed that Ki-67 decreased significantly after treatment with ES-Cu&GOx@FFC, which laid the foundation for improved prognosis. At the same time, ES-Cu&GOx@FFC can promote the upregulation of PD-L1, providing a target for ICB therapy, which can improve the clinical benefit rate of αPD-L1 treatment [48]. In summary, the results of this study indicate that ES-Cu&GOx@FFC can not only perform multi-mechanism synergistic anti-cancer functions, but also demonstrate clinical application value.

### 2.7 ES-Cu&GOx@FFC hydrogel can reprogram the tumor microenvironment and activate anti-tumor immune response

Based on the core role of tumor-infiltrating lymphocytes (TILs) in immunotherapy, we systematically evaluated the regulatory ability of ES-Cu&GOx@FFC on the tumor immune microenvironment of mice [49]. The primary tumor and tumor-draining lymph nodes (TDLNs) tissues of tumor-bearing mice were collected, and the changes in immune cell populations were analyzed by flow cytometry.

**Fig. 7:**
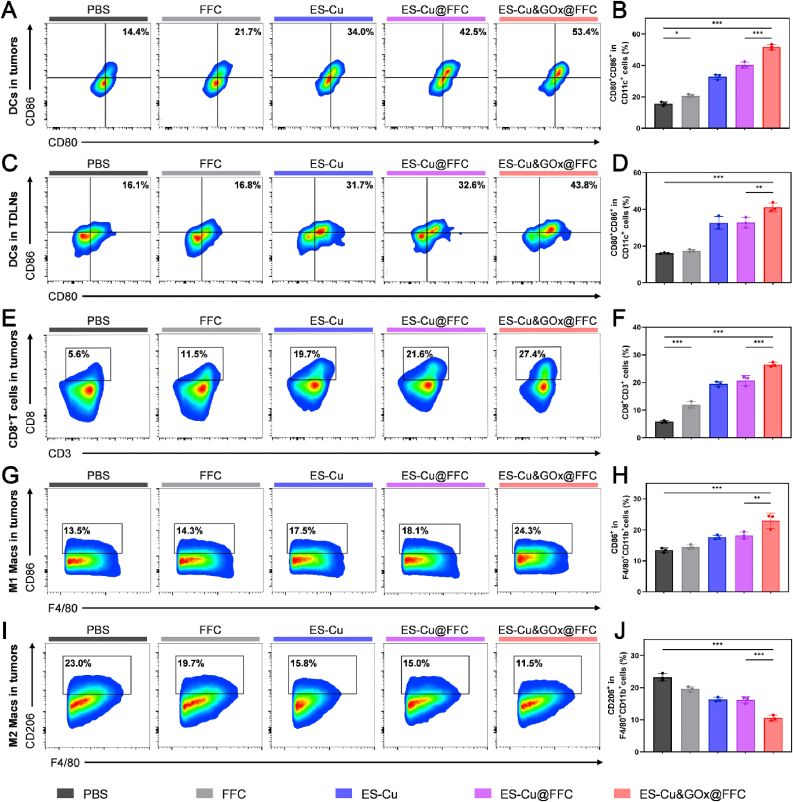
**Reprogramming of TME by ES-Cu&GOx@FFC hydrogel.** (A, B) Flow cytometric analysis and statistics of DCs in tumor tissues of mice after different treatments. (C, D) Flow cytometric analysis and statistics of DCs in TDLNs tissues of mice after different treatments. (E, F) Flow cytometric analysis and statistics of CD8^+^ T cells in tumor tissues of mice after different treatments. (G–J) Flow cytometric analysis and statistics of M1 and M2 macrophages in tumor tissues of mice after different treatments. The indicated results represent the mean ± SD of three independent experiments. ns, not significant, ∗P < 0.05, ∗∗P < 0.01, ∗∗∗P < 0.001.

DCs, as core antigen-presenting cells (APCs), mainly perform the function of capturing tumor-associated antigens and presenting them to T cells, playing a pivotal role in connecting innate and adaptive immunity [50]. Flow cytometry results showed that the proportion of mature DCs (CD80^+^CD86^+^) in tumor tissues of mice treated with ES-Cu&GOx@FFC was 51.7 %, which was 3.3 times that of the group treated with PBS (Fig. 7A and B). The maturation rate of dendritic cells in TDLNs of mice treated with ES-Cu&GOx@FFC was 41.1 %, which was 2.5 times that of the PBS-treated group (Fig. 7C and D). Interestingly, the blank FFC hydrogel itself showed the ability to promote the maturation of DCs in tumor tissues [51], but this phenomenon was not observed in TDLNs. This may be attributed to the immunoadjuvant properties of CS, which directly promoted the maturation of DCs in the tumor, but failed to significantly affect the maturation process of DCs in TDLNs due to the limited lymphatic drainage of the hydrogel material. CD8^+^ T cells, as the core effector cells of the anti-tumor immune response, specifically recognize tumor cells and release perforin, granzyme, and IFN-γ to mediate targeted killing and eliminate cancer cells [52]. Therefore, we studied the infiltration level of CD8^+^ T cells in tumor tissues to analyze the regulatory effect of ES-Cu&GOx@FFC hydrogel on T cells. The results showed that the percentage of CD3^+^ CD8^+^ T cells in tumor tissues of mice treated with ES-Cu&GOx@FFC was 26.5 %, which was 4.6 and 1.3 times that of the PBS and ES-Cu@FFC treatment groups (Fig. 7E and F). These data indicate that the drug delivery system synergistically induces ICD to release DAMPs and promotes DCs maturation, ultimately driving the activation of CD8^+^ T cells to recognize and attack tumor cells. Tumor-associated macrophages (TAMs) are the main immune cell population in the TME, and their phenotypic polarization state directly regulates the tumor immune process. TAMs are mainly divided into pro-inflammatory M1 and pro-tumor M2 types. M1 macrophages (F4/80^+^ CD11b^+^ CD86^+^) can phagocytose cancer cells and recruit T cells; while M2 macrophages (F4/80^+^ CD11b^+^ CD206^+^) can promote tumor growth, invasion, and metastasis. In the TME, TAMs usually exhibit an anti-inflammatory and immunosuppressive M2-like phenotype, which is associated with immune escape and tumor progression. Recent studies have shown that the production of ROS can induce macrophage polarization, causing TAMs to transform from an M2 phenotype to an anti-tumor M1 phenotype, effectively reversing the immunosuppressive microenvironment [53,54]. To explore whether ES-Cu&GOx@FFC can induce the transformation of macrophage phenotype from M2 to M1, we evaluated the content of M1 and M2 TAMs and the ratio of M1/M2 TAMs in tumor tissues. Compared with other treatment groups, ES-Cu&GOx@FFC significantly increased M1 macrophages and decreased M2 macrophages (Fig. 7G–J). The ratio of M1/M2 TAMs in tumor tissues of mice treated with ES-Cu&GOx@FFC was 3.8 times that of PBS control (Fig. S17).

In summary, ES-Cu&GOx@FFC promoted DCs maturation, increased the infiltration ratio of CD8^+^ T cells, drove the polarization of TAMs phenotype from M2 to M1, mediated the reprogramming of TME, and finally reversed the immunosuppressive "cold" tumor microenvironment to the immune-activating "hot" tumor microenvironment.

### 2.8 ES-Cu&GOx@FFC hydrogel combined with αPD-L1 can synergistically enhance the anti-tumor efficacy

In recent years, ICB therapy targeting PD-1 or PD-L1 has become a research focus for tumor treatment. αPD-L1 monoclonal antibody can relieve T cell function inhibition and restore its anti-tumor activity by specifically blocking the binding of PD-L1 to PD-1. However, the therapeutic effect of αPD-L1 is limited by the immunosuppressive TME, failing αPD-L1 alone to achieve the expected effect [55]. This study found that ES-Cu&GOx@FFC can not only induce cuproptosis in 4T1 cells, causing ICD and TME reprogramming, but also upregulate the expression of PD-L1 on the surface of tumor cells. The above results provide a theoretical basis for the combination of αPD-L1, prompting us to further study the synergistic therapeutic strategy of combining ES-Cu&GOx@FFC hydrogel with αPD-L1.

**Fig. 8:**
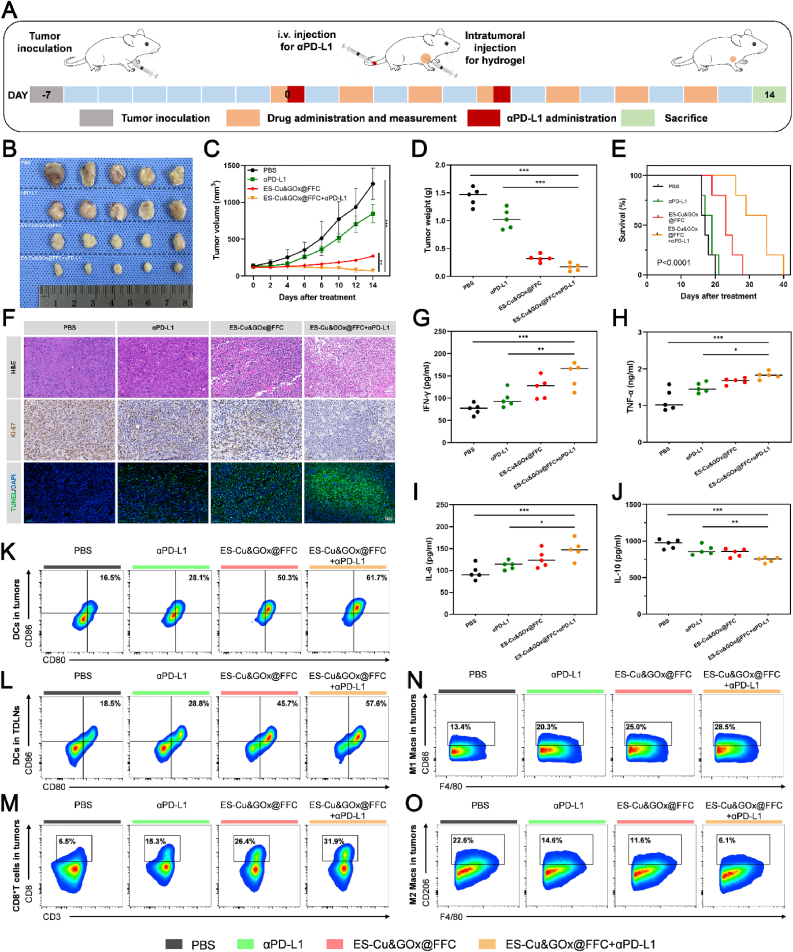
**In vivo antitumor effect of ES-Cu&GOx@FFC hydrogel + αPD-L1 combined therapy.** (A) Schematic diagram of the combined treatment regimen for 4T1 tumor-bearing mice. (B) Photos of dissected tumors on day 14 after different treatments. (C) The curve of subcutaneous tumor volume changes during treatment. (D) Statistical analysis of in vitro tumor weight on day 14 after different treatments. (E) Kaplan–Meier survival analysis of mice receiving different treatments. (F) H&E, Ki-67, and TUNEL staining of in vitro tumor tissues on day 14 after different treatments (Scale bar = 50 μm). (G–J) Concentrations of immune-related cytokines IFN-γ, TNF-α, IL-6, and IL-10 in the serum of mice receiving different treatments. (K–O) Flow cytometric analysis of DCs, CD8^+^ T cells, and M1 and M2 macrophages in tumor tissues or TDLNs of mice receiving different treatments. The indicated results represent the mean ± SD of three or five independent experiments. ns, not significant, ∗P < 0.05, ∗∗P < 0.01, ∗∗∗P < 0.001.

To verify the synergistic effect of combined therapy, we established a 4T1 subcutaneous tumor model in BALB/c mice, and tumor-bearing mice were randomly divided into 4 groups (n = 5) on day 7 after tumor inoculation (referred to as day 0): (1) PBS control group; (2) αPD-L1 monotherapy group; (3) ES-Cu&GOx@FFC group; (4) ES-Cu&GOx@FFC+αPD-L1 combination group. As shown in Fig. 8A, tumors were treated with intratumoral drugs every two days (7 injections in total, with a total dose of 6 mg ES/kg and 151.7 mg GOx/kg). In addition, the immune checkpoint inhibitor αPD-L1 was introduced by intravenous injection on days 0 and 6 (2 injections in total, with a total dose of 10 mg αPD-L1/kg). From the day of drug administration, the tumor volume and body weight of mice were recorded every other day. Compared with the control model group, αPD-L1 administration slightly reduced the tumor growth rate, while the ES-Cu&GOx@FFC+αPD-L1 combined treatment group showed the most significant tumor inhibitory effect. Specifically, the tumor volumes of mice treated with PBS, αPD-L1, ES-Cu&GOx@FFC, and ES-Cu&GOx@FFC+αPD-L1 were 1251.6 mm^3^, 846.0 mm^3^, 268.8 mm^3^, and 72.0 mm^3^, respectively (Fig. 8B, C, Fig. S18). On the 14th day after administration, the mice were euthanized, and the subcutaneous tumors of the mice were collected and weighed. It was found that the ES-Cu&GOx@FFC+αPD-L1 combined treatment group could significantly reduce the weight of the tumor (Fig. 8D). This result is consistent with the tumor volume data, confirming that the combined regimen achieved significant tumor regression through a synergistic mechanism. Encouragingly, we used the same treatment strategy to treat another group of tumor-bearing mice (n = 5). On the 14th day after administration, the mice were not euthanized but continued to be raised, and their survival status was recorded. The results showed that the ES-Cu&GOx@FFC+αPD-L1 combined treatment group could significantly prolong the survival time of mice (Fig. 8E). Next, we performed H&E, Ki-67, and TUNEL staining analysis on the tumor tissues collected after treatment in different treatment groups (Fig. 8F). The results showed that the ES-Cu&GOx@FFC+αPD-L1 combined treatment group showed more significant nuclear fragmentation and nuclear dissolution, lower Ki-67 positivity rate, and more tumor necrosis or apoptosis compared with the single-drug group and the control group. The above results show that the anti-tumor effect of the ES-Cu&GOx@FFC+αPD-L1 combined treatment group is stronger than that of αPD-L1 alone or ES-Cu&GOx@FFC alone, proving that ES-Cu&GOx@FFC sensitizes the efficacy of αPD-L1, and the two play a synergistic effect during the treatment process. To systematically evaluate the immune activation effect of combined therapy, we quantitatively analyzed the concentrations of immune-related cytokines interferon-*γ* (IFN-γ), tumor necrosis factor-α (TNF-α), interleukin-6 (IL-6), and interleukin-10 (IL-10) in mouse serum by ELISA. The results showed that after combined treatment with ES-Cu&GOx@FFC+αPD-L1, the levels of IFN-γ, TNF-α, and IL-6 were increased, and the level of IL-10 was reduced (Fig. 8G–J). This confirms that the combined treatment strategy activates anti-tumor immune response by reshaping the cytokine network. To further explore the effect of ES-Cu&GOx@FFC+αPD-L1 on TME, we collected tumor and TDLNs tissues from mice in different treatment groups and analyzed the relevant immune parameters using flow cytometry. The results of flow cytometry showed that the proportion of mature DCs in the tumor tissues of mice treated with ES-Cu&GOx@FFC+αPD-L1 reached 58.4 %, which was 2.1 times and 1.2 times that of the αPD-L1 and ES-Cu&GOx@FFC treatment groups, respectively (Fig. 8K, Fig. S19). The maturation rate of dendritic cells in TDLNs of mice treated with ES-Cu&GOx@FFC+αPD-L1 was 54.7 %, which was significantly higher than 27.2 % and 43.2 % in the αPD-L1 and ES-Cu&GOx@FFC monotherapy groups (Fig. 8L, Fig. S20). In addition, the ES-Cu&GOx@FFC+αPD-L1 combined treatment group also increased the infiltration of CD8^+^T cells in the TME. Compared with the αPD-L1 and ES-Cu&GOx@FFC treatment groups, the infiltration ratio of CD8^+^T cells increased from 15.9 % to 25.1 %–34.3 % (Fig. 8M, Fig. S21). Finally, we explored the polarization of TAMs. Compared with the monotherapy group, TAMs in the ES-Cu&GOx@FFC+αPD-L1 combined treatment group showed an increase in the M1 type and a decrease in the M2 type (Fig. 8N, O, Figure S22, 23). Meanwhile, the combined treatment of the drug-loading platform and immune checkpoint inhibitors significantly increased the ratio of M1/M2, which was 2.3 and 4.8 times that of the ES-Cu&GOx@FFC group and αPD-L1 group (Fig. S24).

In summary, our multidimensional experimental results confirmed that ES-Cu&GOx@FFC+αPD-L1 therapy promoted anti-tumor efficacy by effectively reprogramming the immunosuppressive TME.

### 2.9 Biosafety assessment

Based on the excellent in vivo antitumor efficacy of the ES-Cu&GOx@FFC hydrogel, we conducted further biosafety assessments, including hemolysis experiments, mouse body weight changes, routine blood count parameters, liver and kidney biochemical markers, and pathological morphology of major organs. As shown in Fig. S25, the hemolysis assay showed that even with a drug loading of 5 times the pre-designed dose, the hemolysis rate remained below the internationally recognized standard (5 %), preliminarily demonstrating the in vivo safety of the ES-Cu&GOx@FFC hydrogel. The body weight change data showed that the body weights of mice treated with different methods changed during the treatment period, and there was no statistically significant difference among the groups (Fig. S26). Furthermore, routine blood counts and biochemical parameters in mice in all treatment groups, including RBC count, PLT count, ALT, AST, BUN, and CREA, were all within normal ranges, demonstrating that in situ ES-Cu&GOx@FFC treatment did not induce hematological abnormalities or hepatotoxicity or renal toxicity (Fig. S27A–F). Given the presence of Cu and GOx loaded into the ES-Cu&GOx@FFC hydrogel, we further investigated whether these drugs would affect serum copper concentrations and blood glucose in mice. The results showed that serum copper concentrations and blood glucose remained within normal ranges in all treatment groups (Fig. S27G and H). H&E staining of major organs is shown in Fig. S28. Compared to the control group, no significant morphological changes were observed in the heart, liver, spleen, lung, and kidneys after treatment in all treatment groups. The excellent biosafety exhibited by ES-Cu&GOx@FFC is premised on the fact that the components of the gel are all FDA-approved pharmaceutical excipients, which can avoid the short-term or long-term toxicity of traditional industrially produced synthetic nanomaterials. In summary, ES-Cu&GOx@FFC did not cause systemic toxicity at highly effective antitumor doses and has potential for clinical translation.

## 3 Conclusion

In summary, this study demonstrates the unique therapeutic potential of cuproptosis in TNBC and synthesizes an injectable smart hydrogel for the local delivery of ES-Cu and GOx to enhance the therapeutic effect of αPD-L1. The encapsulated ES-Cu and GOx are gradually released, killing tumors by inducing cuproptosis, starvation therapy, and CDT. ES-Cu&GOx@FFC can also induce cell release of DAMPs, further triggering an anti-tumor immune response. In vitro data demonstrate that ES-Cu&GOx@FFC promotes DCs maturation, increases CD8^+^ T cell infiltration, and induces macrophage polarization to an M1-like phenotype, effectively reversing the tumor's immunosuppressive microenvironment. Specifically, in an in vivo TNBC tumor model, peritumoral injection of ES-Cu&GOx@FFC monotherapy achieved >80 % tumor regression rates. Combining ES-Cu&GOx@FFC with αPD-L1 further activated the anti-tumor immune response, increasing the tumor regression rate to over 90 % and significantly improving survival outcomes. The smart drug-loaded hydrogel designed in this study provides a new solution for sensitizing TNBC immunotherapy by resolving the heterogeneity of PD-L1 and overcoming the "cold tumor" immune microenvironment. At the same time, ES-Cu&GOx@FFC also has exciting translational potential. This smart hydrogel is synthesized using clinically friendly materials, and the synthesis steps are simple, which reduces the translation cost to a certain extent and can also reduce the economic burden on cancer patients. However, as TNBC research deepens, researchers have proposed more detailed TNBC classification strategies. In future studies, we hope to further optimize the design and apply a variety of cell and animal models to investigate the responses of different TNBC classifications to treatment. In general, this injectable ES-Cu&GOx@FFC smart dual-responsive hydrogel provides a new solution for TNBC that is a multi-mechanism synergistic anti-cancer material with the ability to reprogram the immune microenvironment and has clinical translation potential, opening up a new path to overcome the bottleneck of TNBC efficacy.

## 4 Experimental section

### 4.1 Materials and reagents

Elesclomol (ES) and Glucose oxidase (GOx) were purchased from MCE (USA), chitosan (CS) was purchased from Solarbio (Beijing, China), and F127 and F68 were purchased from Yuanye Biotechnology (Shanghai, China). In vivo mAb anti-mouse PD-L1 was purchased from Bio X Cell (USA). DLAT antibody, FDX1 antibody, LIAS antibody, GAPDH antibody, CRT antibody, HMGB1 antibody, PD-L1 antibody, and other western blotting, immunohistochemistry, and immunofluorescence related antibodies were purchased from Proteintech (Wuhan, China). Anti CD45-PE, anti CD274-APC, anti CD11c-APC, anti CD80-BV421, anti CD86-PECY7, anti CD3-APC, anti CD4-FITC, anti CD8-PECY7, anti CD45-BV510, anti CD11b-PECY7, anti F4/80-PE, anti CD86-APC, anti CD206-FITC, and other flow cytometry-related antibodies were purchased from Biolegend (USA). 1640 culture medium, fetal bovine serum, and penicillin/streptomycin double antibody were purchased from GIBCO (USA).

### 4.2 Cell lines and animals

For in vitro experiments, the human breast cancer cell line MDA-MB-231 and the murine mammary adenocarcinoma cell line 4T1 were purchased from Abcell (Beijing, China). All cells were cultured in DMEM or 1640 medium supplemented with 10 % fetal bovine serum and 1 % penicillin/streptomycin. The incubator temperature was maintained at 37 °C with 5 % CO_2_. The MDA-MB-231 cell line was used to analyze transcriptome changes after cuproptosis, and the 4T1 cell line was used for biological validation of the hydrogel drug delivery platform. For in vivo experiments, female BALB/c mice (6–8 weeks old) were purchased from the Animal Experimental Center of Harbin Medical University and maintained under specific pathogen-free conditions. 4T1 cells were inoculated subcutaneously in the right axilla of BALB/c mice to establish a subcutaneous breast cancer xenograft model. This model was used for in vivo antitumor and immunotherapy studies of the hydrogel drug delivery platform. Animal experiments were approved by the Ethics Committee of the Second Affiliated Hospital of Harbin Medical University.

### 4.3 RNA-seq and bioinformatics analysis

The total RNA of 4T1 cells was extracted according to the instructions of the TRIzol reagent. RNA-seq was performed by IGENEBOOK (Wuhan, China), and the raw image data files obtained by high-throughput sequencing were converted into raw sequencing sequences by base recognition for subsequent analysis. The filtering conditions for differential genes were set as p-value <0.05 and |Log_2_FC|≥1. R language and the Hiplot online tool (https://hiplot.com.cn/) were used to visualize differential gene heat maps, volcano maps, KEGG enrichment, and GO enrichment. According to previous studies, 16 cuproptosis, 10 immunogenic death, and 7 immune checkpoint-related core genes were obtained. The relationship between DLAT expression and immune cell infiltration in TNBC was analyzed using the TIMER algorithm.

### 4.4 Clinical tissue sample

Clinical tissue specimens were collected from patients who underwent surgery in the Department of Breast Surgery, the Second Affiliated Hospital of Harbin Medical University, from September 2022 to September 2023. A total of 10 TNBC patients were collected. All patients were diagnosed with triple-negative breast cancer by histopathological examination, excluding other malignant tumors, and no major organ diseases. All tissues were snap-frozen in liquid nitrogen immediately after surgical resection until protein extraction. This study was approved by the Ethics Committee of the Second Affiliated Hospital of Harbin Medical University, and written informed consent was obtained from all patients.

### 4.5 Preparation of FFC hydrogel and ES-Cu&GOx@FFC hydrogel

To prepare the FFC hydrogel, chitosan was first added to glacial acetic acid to create a 2 % chitosan solution (w/v). After complete dissolution under magnetic stirring, F68 and F127 were added to the chitosan solution and magnetically stirred for 5 min to mix thoroughly. The solution was then stored in a refrigerator at 4 °C overnight to allow for full swelling and dissolution, thus preparing the FFC solution. The FFC solution was heated in a 37 °C water bath for 3–5 min. A gel was considered formed when the liquid ceased to flow after inverting the centrifuge tube for 30 s. In optimizing the concentration of the key component responsible for the hydrogel's thermosensitivity, experiments revealed that when the F127 concentration was below 25 %, the system failed to form a stable gel network at 37 °C, while a concentration exceeding 30 % made it difficult to dissolve. Therefore, three F127 concentration gradients within the effective F127 concentration range in the FFC hydrogel were selected: 30 %, 27.5 %, and 25 %, for further thermosensitivity optimization studies. Temperature sweep rheological tests found that the FFC hydrogel carrier with a 25 % F127 concentration exhibited the most suitable LCST (33.5 °C) for in situ gelation in the body. Finally, we used FFC hydrogels with a final concentration of 6 % (w/v) of F68 and a final concentration of 25 % of F127 for subsequent synthesis.

For the preparation of ES-Cu&GOx@FFC hydrogel, DMSO was first used as a solvent to prepare ES stock solution (1 mmol/L), and deionized water was used to prepare CuCl_2_ stock solution (1 mol/L) and GOx stock solution (1 mg/mL). 40 μL of ES, 40 μL of CuCl_2_, and 400 μL of GOx were added to 20 mL of FFC solution, and the mixed solution was vortexed for 10 s to obtain FFC solution loaded with ES-Cu and GOx. The ES-Cu&GOx@FFC solution was placed in a water bath and heated for 3 min to prepare ES-Cu&GOx@FFC hydrogel. For animal experiments, the final concentration of ES in ES-Cu&GOx@FFC hydrogel was 160 μM and the final concentration of GOx was 1.6 mg/mL. To enhance the visual performance of ES-Cu&GOx@FFC hydrogel formation, we introduced an appropriate amount of methylene blue into the mixture before photography.

### 4.6 Scanning electron microscopy (SEM)

The internal morphology of FFC and ES-Cu&GOx@FFC hydrogels was examined by SEM. After quick freezing and freeze drying, the hydrogels were brittle fractured in liquid nitrogen to expose the internal region, sputter-coated with gold, and the internal structure was examined in a microscope (ZEISS GeminiSEM 300).

### 4.7 Rheological test

A TA rheometer (DHR-2) was used to evaluate the rheological properties of the hydrogels. The hydrogels to be tested were placed between parallel plates with a diameter of 20 mm to detect the storage modulus (G’) and loss modulus (G”). The temperature ramp test was performed with a frequency of 1 Hz, a strain of 1 %, and a temperature range of 0–45 °C. The time sweep test was performed with a frequency of 1 Hz, a strain of 1 %, and a temperature of 37 °C. The frequency test was performed with a strain of 1 %, a temperature of 37 °C, and a frequency range of 0.01–100 Hz.

### 4.8 pH responsiveness

ES-Cu&GOx@FFC hydrogels were placed in 12-well plates under incubation conditions at 37 °C, and PBS buffers with different pH values were added as dissolution media. PBS was collected at designated time points, and free ES in the supernatant was quantified by HPLC (Agilent HPLC 1260). For the cumulative release of GOx, we used a fixed amount of glucose as a substrate and reflected the cumulative release of GOx by the consumption of glucose.

### 4.9 Enzyme activity assay

To verify the glucose catalysis and H_2_O_2_ generation ability of ES-Cu&GOx@FFC, a 10 mM glucose solution was mixed with ES-Cu&GOx@FFC in equal volumes, and the solution was collected. The glucose or H_2_O_2_ concentration was determined using a glucose or H_2_O_2_ content determination kit (Solarbio, Beijing, China) according to the kit instructions.

The GSH consumption-ability of ES-Cu&GOx@FFC was detected by the reaction of DTNB with GSH. Different experimental groups were mixed with a 3 mM GSH solution in equal volumes and reacted for 1 h. The GSH concentration was determined by measuring the absorbance of the yellow product at 415 nm using a GSH content determination kit (Solarbio, Beijing, China).

The •OH generation ability was evaluated by measuring the absorbance of the colored product generated by different experimental groups in the presence of glucose or H_2_O_2_ alone at 532 nm using an •OH content kit (Aidisheng, China).

For copper content determination, different experimental groups were placed in 12-well plates at 37 °C, and 9 vol of PBS buffer were added as the dissolution medium. The supernatant was collected after 1 h, and the free Cu in the supernatant was quantified using a copper content kit.

### 4.10 Determination of intracellular copper content

4T1 cells were seeded in 6-well plates (5 × 10^5^ cells per well) and incubated overnight. Then PBS, FFC (200 μL), ES-Cu (ES final concentration in the culture medium was 200 nM), ES-Cu@FFC (200 μL), and ES-Cu&GOx@FFC (200 μL) were added for 24 h, and then the cells were washed three times with PBS. The cells were collected, and the intracellular Cu content was determined using a cell Cu content detection kit (Solarbio, Beijing, China) according to the manufacturer's instructions.

### 4.11 Cell viability assay

4T1 cells were seeded into 96-well plates (5000 cells per well) and incubated overnight. Then PBS, FFC (10 μL), ES-Cu (ES final concentration in the culture medium was 200 nM), ES-Cu@FFC (10 μL), and ES-Cu&GOx@FFC (10 μL) were added for 24 h. The culture medium was then discarded. 90 μl of 1640 culture medium and 10 μl of CCK-8 solution (SEVEN BIOTECH, Beijing, China) were added to each well and incubated for another 2 h. The absorbance at 450 nm was measured using a microplate reader (Bio-Rad, USA).

### 4.12 Cell morphology

4T1 cells were seeded in 6-well plates (5 × 10^5^ cells per well) and incubated overnight. Then PBS, FFC (200 μL), ES-Cu (ES final concentration in the culture medium was 200 nM), ES-Cu@FFC (200 μL), and ES-Cu&GOx@FFC (200 μL) were added for 24 h. The morphology of the cells was further observed using an inverted microscope (ZEISS, Germany).

### 4.13 Apoptosis assay

4T1 cells were seeded in 6-well plates (5 × 10^5^ cells per well) and incubated overnight. Then PBS, FFC (200 μL), ES-Cu (ES final concentration in the culture medium was 200 nM), ES-Cu@FFC (200 μL), and ES-Cu&GOx@FFC (200 μL) were added for 24 h. The collected cells were stained with Annexin V-FITC and PI apoptosis detection kits (SEVEN BIOTECH, Beijing, China) and analyzed by flow cytometry (Apogee, UK).

### 4.14 Western blot

Cell samples from different treatment groups were collected and lysed using RIPA lysis. Proteins were loaded onto SDS-PAGE gels and transferred to PVDF membranes (Millipore, USA) after electrophoresis. After blocking with skim milk powder, the membranes were incubated with primary antibodies at 4 °C overnight and with secondary antibodies at room temperature for 1 h. Chemiluminescent detection reagents (Beyotime, Shanghai, China) were used for exposure and development. The experimental results were analyzed and quantified using ImageJ software.

### 4.15 Intracellular ROS and GSH detection

Flow cytometry and fluorescence microscopy were used to detect intracellular ROS levels. Briefly, 4T1 cells were seeded in 6-well plates, and then the cells were treated with different treatment groups for 24 h. Then the cells were incubated with 10 μM DCFH-DA at 37 °C for 30 min according to the manufacturer's instructions. After removing DCFH-DA, the cells were washed three times with DMEM, and fluorescence images were taken using a fluorescence microscope (Nikon, Japan) or analyzed by flow cytometry.

To detect intracellular GSH, cells from different treatment groups were collected, and the intracellular GSH level was determined using a GSH content assay kit according to the manufacturer's instructions.

### 4.16 Immunofluorescence detection of DLAT, HMGB1, and CRT

4T1 cells were seeded in 6-well plates (2 × 10^5^ cells per well) and incubated overnight, and then treated with different treatment groups for 24 h. The cells were fixed with 4 % PFA, followed by cell membrane permeabilization, blocking, incubation with primary antibodies, incubation with fluorescent secondary antibodies, and DAPI staining of cell nuclei. Finally, fluorescence images were taken using a fluorescence microscope.

### 4.17 In vivo anti-tumor effect and biosafety experiments

4T1 cells were injected subcutaneously into BALB/c mice (1x106 cells per mouse). After the subcutaneous tumors grew for one week, the mice were randomly divided into 5 groups. Different experimental group drugs were injected into the mouse tumors in batches. The tumor volume and body weight of the mice were recorded every two days. The formula for calculating tumor volume is: volume (mm^3^) = longest diameter × shortest diameter^2^/2. The mice were killed on the 14th day, and the final tumor weight was recorded. The serum of the mice was collected for routine blood tests and blood biochemical tests. Tumors and major organs (heart, liver, spleen, lungs, and kidneys) were collected and fixed with 4 % universal tissue fixative, then embedded, tissue sections were obtained, and H&E staining was performed. Tumor sections were further analyzed by Ki-67, FDX1, LIAS immunohistochemical staining, and TUNEL immunofluorescence detection.

Flow cytometry analysis: The mice were killed on the 14th day, tumor tissues were obtained, the tumors were cut into small pieces, and digested on a shaker at 37 °C for 45–60 min to obtain single-cell suspensions. Tumor-infiltrating lymphocytes were extracted using a lymphocyte isolation kit (Solarbio, Beijing, China), and the lymphocyte suspension was then stained with the corresponding fluorescent dye-conjugated antibody in the dark for 30 min. Flow cytometry analysis was performed, and the results were analyzed using FlowJo software.

### 4.18 In vivo anti-tumor effect of combined αPD-L1

The mouse tumor-bearing model was established as described above. On day 0 (7th day after tumor cell injection), mice were randomly divided into four groups: PBS group, αPD-L1 group, ES-Cu&GOx@FFC group, and ES-Cu&GOx@FFC+αPD-L1 group. The tumor volume and body weight of mice were recorded every two days. After the mice were killed on day 14, the final tumor weight was recorded. Tumor tissues were cut, fixed, and embedded in paraffin, and H&E staining, Ki-67 immunohistochemistry, and TUNEL immunofluorescence detection were performed, respectively. Mouse serum was collected for the detection of immune-related cytokines. Mouse tumors were collected, and tumor-infiltrating immune cells were detected.

The same modeling method and administration strategy were used, but the mice were not killed on day 14 to study the survival of the mice.

### 4.19 Statistical analysis

Data are presented as the mean ± standard deviation (SD) of at least three independent experiments. Statistical analysis was performed using GraphPad Prism 9.5 (GraphPad, USA). Comparisons between two groups were analyzed using the T-test. Comparisons between multiple groups were performed using one-way analysis of variance when normal distribution was observed; those not normally distributed were analyzed using the rank-sum test. Survival data were compared using the log-rank test. Results were considered statistically significant when the P value was less than 0.05 (∗, P < 0.05; ∗∗, P < 0.01; ∗∗∗, P < 0.001).

## CRediT authorship contribution statement

**Baiyang Fu:** Writing – original draft, Validation, Methodology, Data curation, Conceptualization. **Guangyan Li:** Writing – original draft, Visualization, Conceptualization. **Yuan Yao:** Writing – original draft, Software, Conceptualization. **Mingfu Zhang:** Software, Conceptualization. **Yesheng Zhong:** Project administration, Methodology. **Xi Wang:** Validation, Methodology. **Yichi Chen:** Validation, Methodology. **Wenlong Liang:** Supervision, Formal analysis. **Yao Wang:** Visualization, Investigation. **Haiyun Lin:** Validation, Methodology. **Yutong Zhang:** Investigation, Formal analysis. **Qiguang Du:** Investigation, Formal analysis. **Zhongkai Xu:** Formal analysis, Data curation. **He Cui:** Investigation, Data curation. **Liping Shi:** Writing – review & editing, Supervision. **Xi Chen:** Writing – review & editing, Supervision, Project administration. **Jianguo Zhang:** Writing – review & editing, Supervision, Project administration.

## Patient consent statement

All patients provided informed consent.

## Ethics approval statement

The Ethics Committee of the Second Affiliated Hospital of Harbin Medical University approved all aspects of this study.

## Permission to reproduce material from other sources

Not applicable.

## Clinical trial registration

Not applicable.

## Funding statement

This work was supported by Beijing Dadi Medical Charity Foundation (No. DDYL-A-KT-20241104-0099).

## Declaration of competing interest

The authors declare that they have no known competing financial interests or personal relationships that could have appeared to influence the work reported in this paper.

## Data Availability

Data will be made available on request.

## Abbreviations

triple-negative breast cancer: (TNBC)
immune checkpoint blockade: (ICB)
programmed death ligand 1: (PD-L1)
tumor microenvironment: (TME)
immunogenic cell death: (ICD)
elesclomol: (ES)
glucose oxidase: (GOx)
chemodynamic therapy: (CDT)
reactive oxygen species: (ROS)
immunogenic cell death: (ICD)
damage-associated molecular patterns: (DAMPs)
dendritic cells: (DCs)
lower critical solution temperature: (LCST)
enzyme-linked immunosorbent assay: (ELISA)
tumor-infiltrating lymphocytes: (TILs)
tumor-draining lymph nodes: (TDLNs)
antigen-presenting cells: (APCs)
tumor-associated macrophages: (TAMs)
