# Supplementary material for: Clinically friendly smart hydrogel boosts cuproptosis and PD-L1 upregulation to enhance anti-tumor immunotherapy
Source: Mater Today Bio. 2025 Sep 9;35:102268. doi: 10.1016/j.mtbio.2025.102268 (PMC12859559; doi:10.1016/j.mtbio.2025.102268)
Supplement: Multimedia component 1 [file mmc1.docx]

Supporting information

**Clinically Friendly Smart Hydrogel Boosts Cuproptosis and PD-L1 Upregulation to Enhance Anti-Tumor Immunotherapy**

Baiyang Fu^#1^, Guangyan Li^#1^, Yuan Yao^#2^, Mingfu Zhang^3^, Yesheng Zhong^3^, Xi Wang^1^, Yichi Chen^4^, Wenlong Liang^1^, Yao Wang^1^, Haiyun Lin^1^, Yutong Zhang^1^, Qiguang Du^1^, Zhongkai Xu^1^, He Cui^1^, Liping Shi*^3^, Xi Chen*^1^, Jianguo Zhang*^1^

^1^ Department of Breast Surgery, The Second Affiliated Hospital of Harbin Medical University, No. 246 Xuefu Road, Harbin, 150000, China.

^2^ Department of Ultrasound, The Second Affiliated Hospital of Harbin Medical University, No. 246 Xuefu Road, Harbin, 150000, China.

^3^ National Key Laboratory of Science and Technology on Advanced Composites in Special Environments, Harbin Institute of Technology, No. 92 Xidazhi Street, Harbin, 150000, China.

^4^ Department of Ultrasound, Harbin Medical University Cancer Hospital, No. 150 Haping Road, Harbin, 150000, China.

^#^B.F., G.L., and Y.Y. contributed equally to this work.

*Corresponding author:J.Z., zhangjianguo27@126.com; X.C., 13804517666@163.com; L.S., shiliping@hit.edu.cn

**
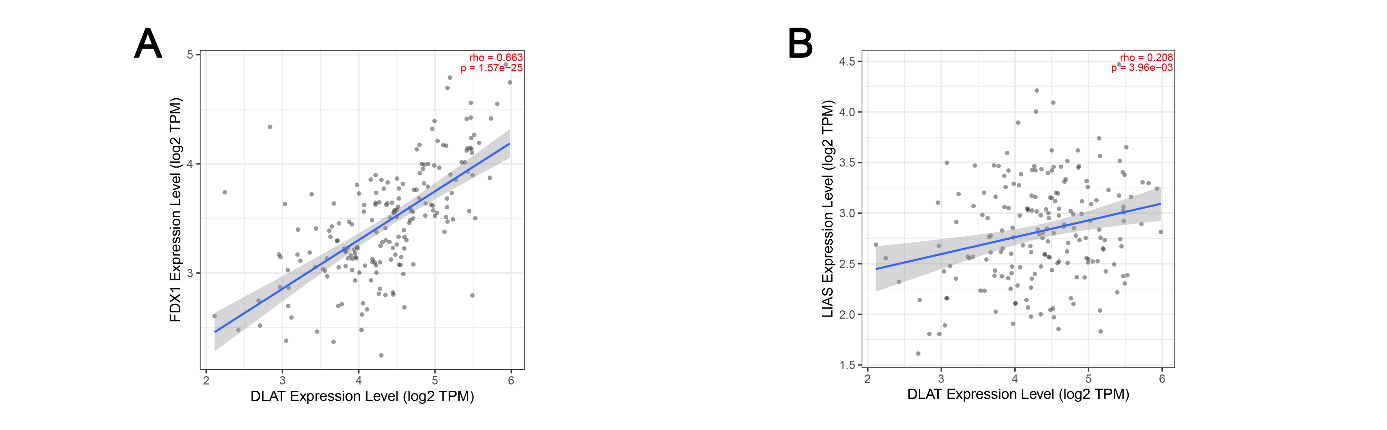
**

**Figure S1.** (A) Correlation between mRNA expression of DLAT and FDX1 in TNBC. (B) Correlation between mRNA expression of DLAT and LIAS in TNBC.

**
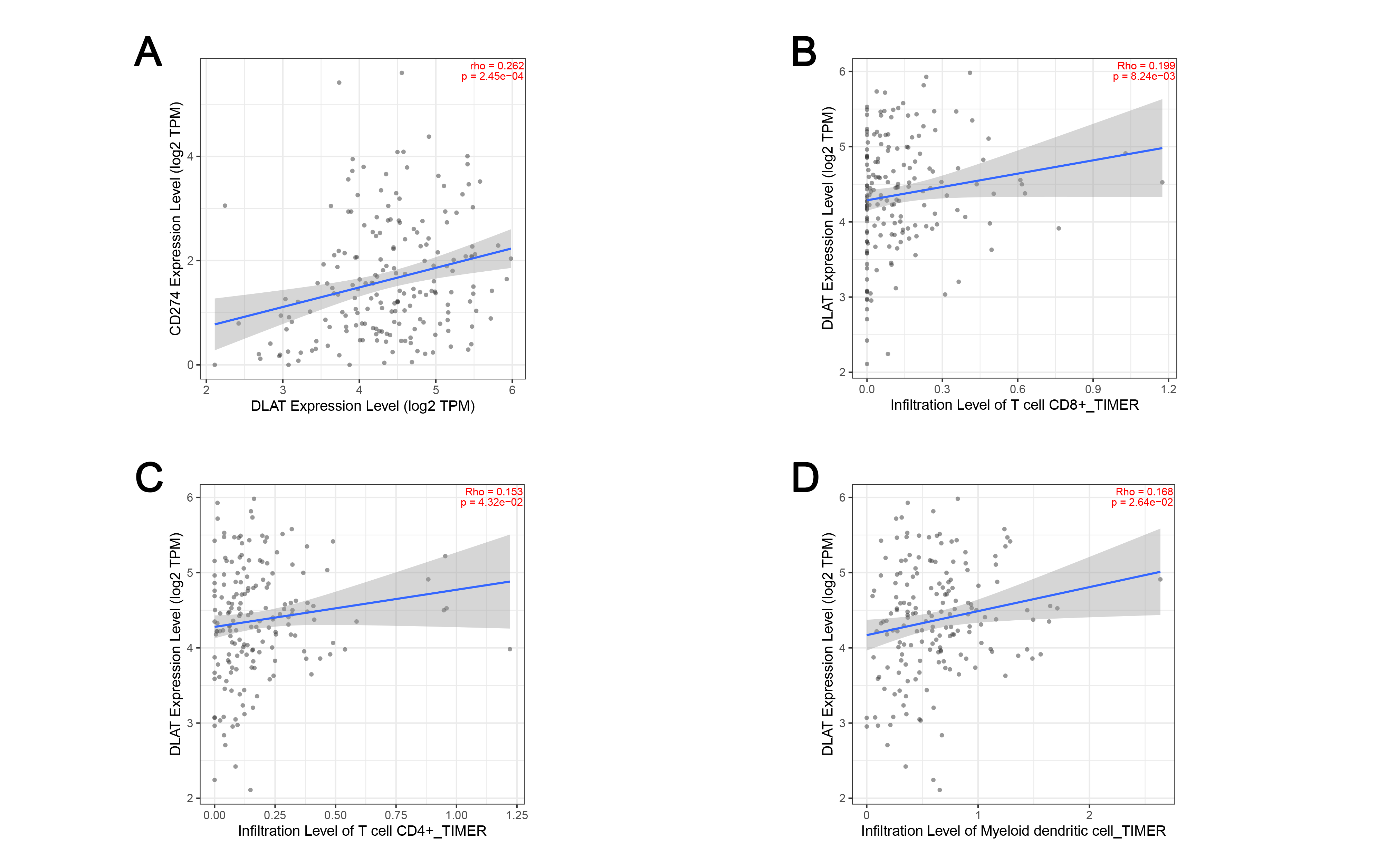
**

**Figure S2.** (A) Correlation between mRNA expression of DLAT and PD-L1 (CD274) in TNBC. (B-D) Correlation between DLAT and infiltration abundance of CD4^+^ T cells, CD8^+^ T cells, and DCs in TNBC based on the TIMER algorithm.

**
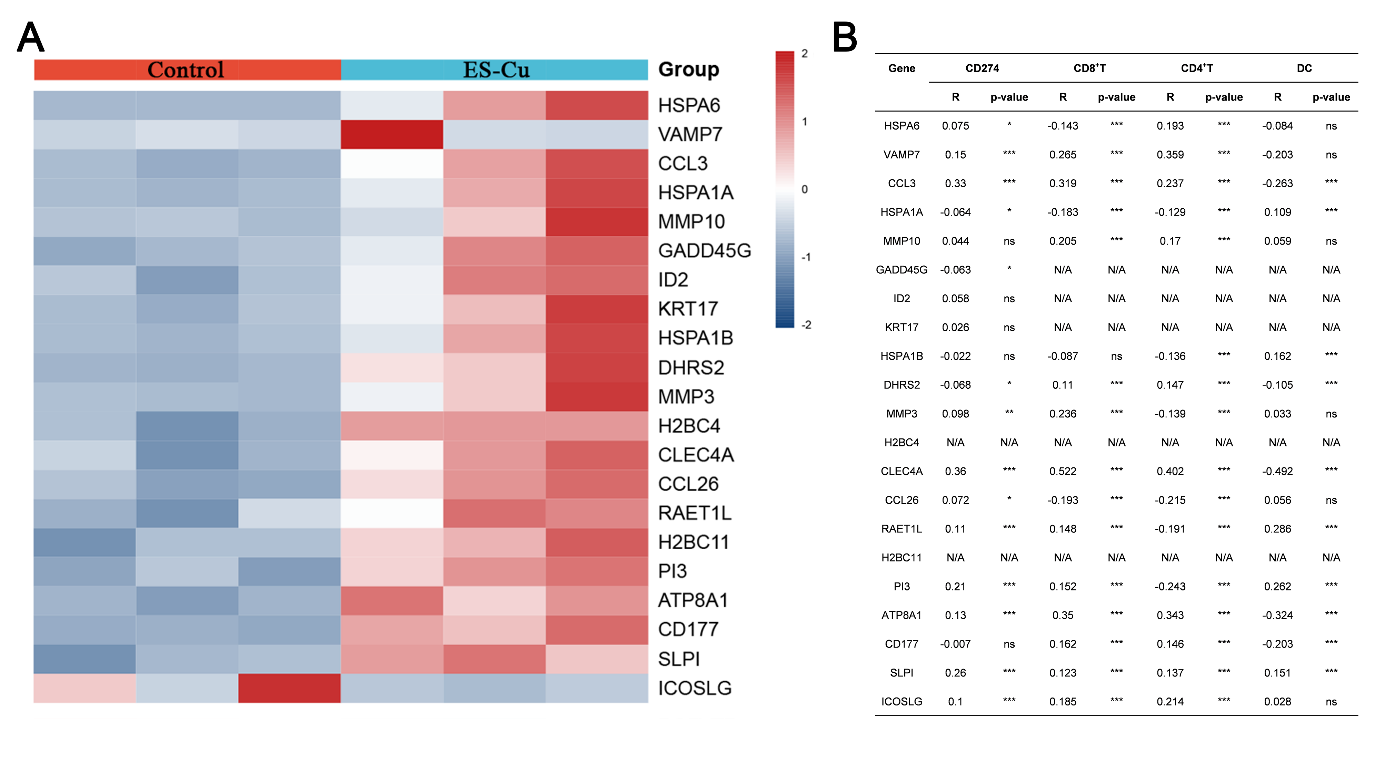
**

**Figure S3.** (A) Heatmap depicts transcriptome changes of “immune response” and “leukocyte activation” related genes after cuproptosis induction. (B) Correlation analysis between “immune response” and “leukocyte activation” related genes and CD274, CD8^+^T cells infiltration, CD4^+^T cells infiltration, and DCs infiltration. ns, not significant, *P < 0.05, **P < 0.01, ***P < 0.001.

**
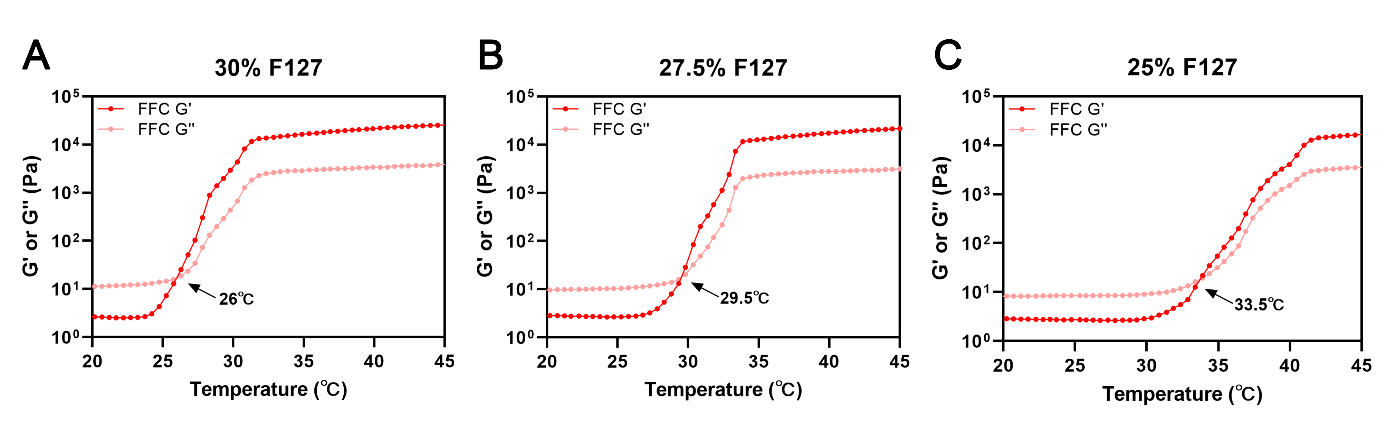
**

**Figure S4.** (A-C) Evolution of G’ and G” of FFC hydrogels with different F127 concentrations in the range of 20-45℃.

**
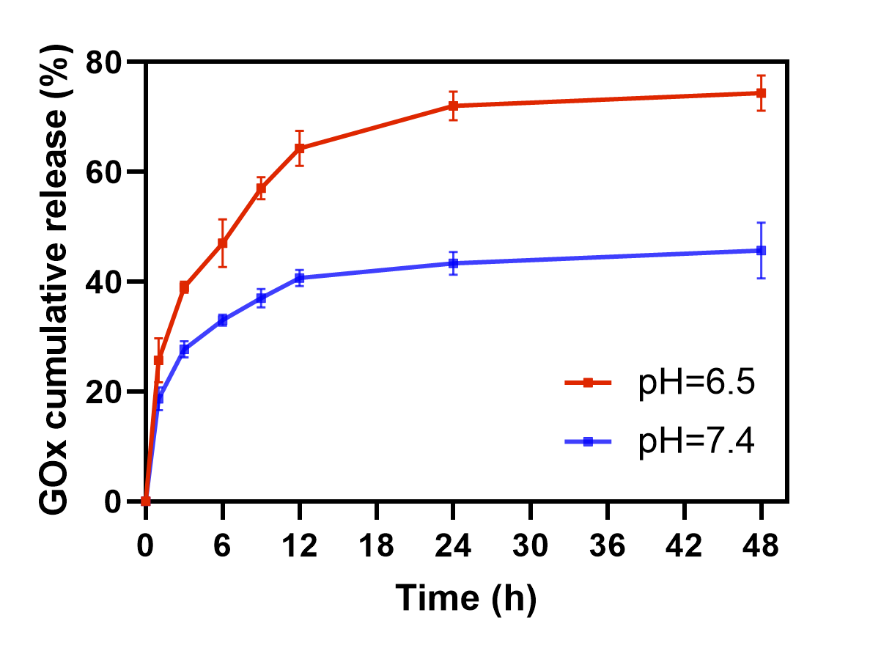
**

**Figure S5.** Cumulative release of GOx from ES-Cu&GOx@FFC gels in buffer solutions of different pH. The indicated results represent the mean ± SD of three independent experiments.

**
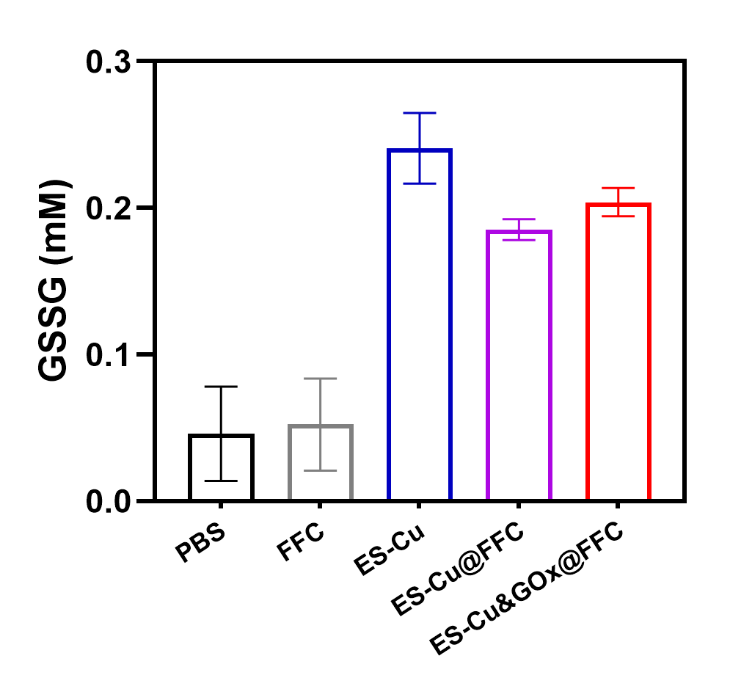
**

**Figure S6.** GSSG content after incubation of different treatment groups. The indicated results represent the mean ± SD of three independent experiments.

**
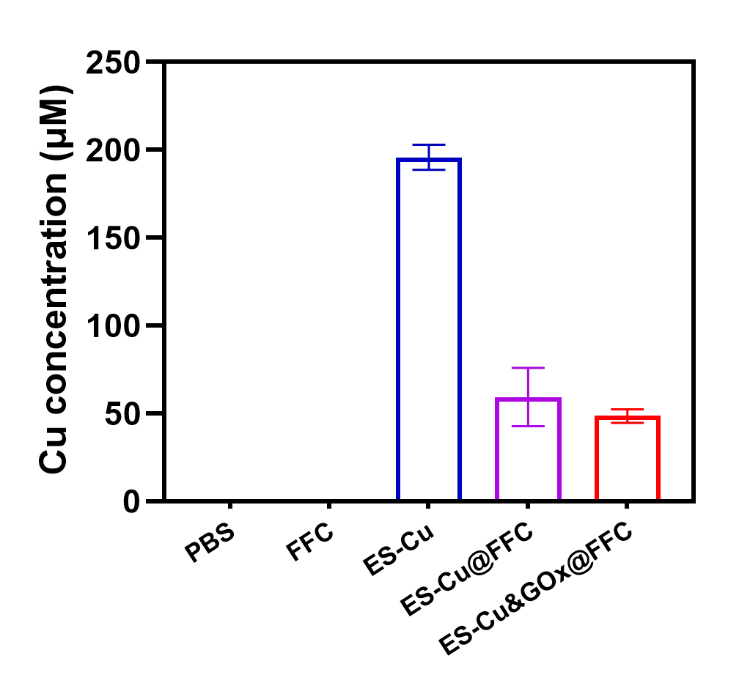
**

**Figure S7.** Cu content in the supernatant of different treatment groups. The indicated results represent the mean ± SD of three independent experiments.

**
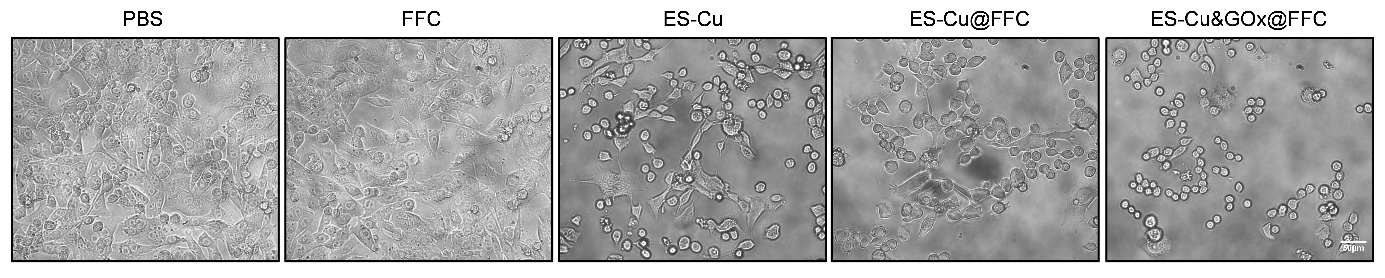
**

**Figure S8.** Representative morphological changes of 4T1 cells after treatment with different treatment groups (Scale bar = 50μm).

**
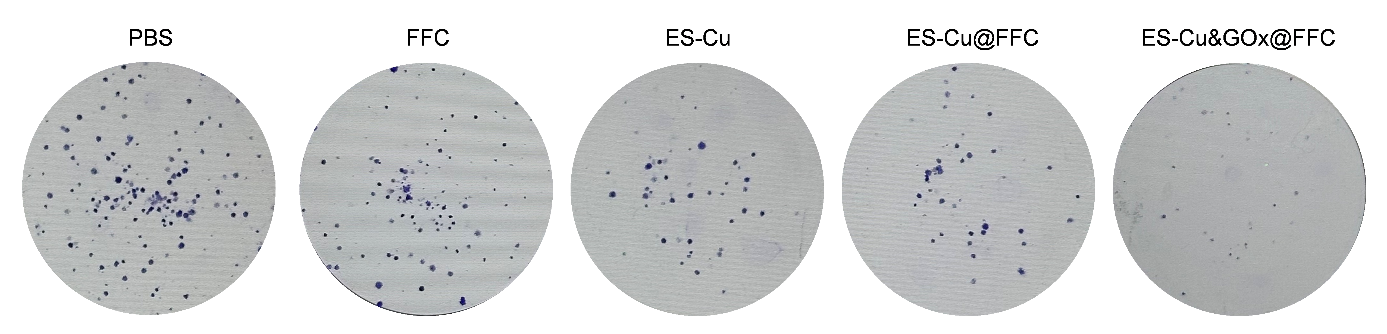
**

**Figure S9.** Number of colony formation of 4T1 cells after treatment with different treatment groups.

**
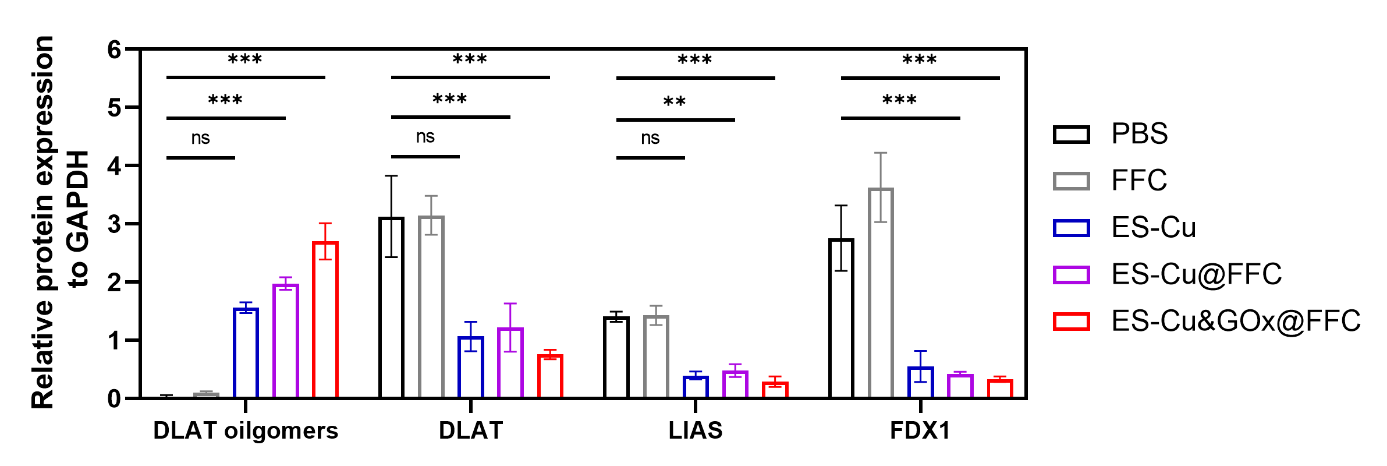
**

**Figure S10.** Quantitative analysis of cuproptosis-related proteins expression in 4T1 cells of different treatment groups after normalization with GAPDH. The indicated results represent the mean ± SD of three independent experiments. ns, not significant, *P < 0.05, **P < 0.01, ***P < 0.001.

**
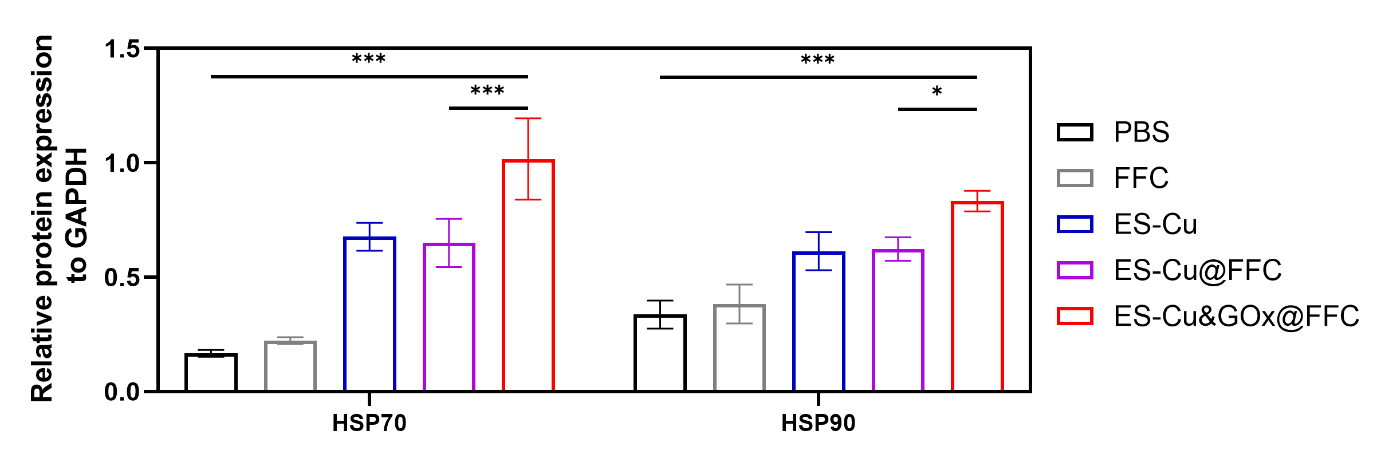
**

**Figure S11.** Quantitative analysis of heat shock proteins expression in 4T1 cells of different treatment groups after normalization with GAPDH. The indicated results represent the mean ± SD of three independent experiments. ns, not significant, *P < 0.05, **P < 0.01, ***P < 0.001.

**
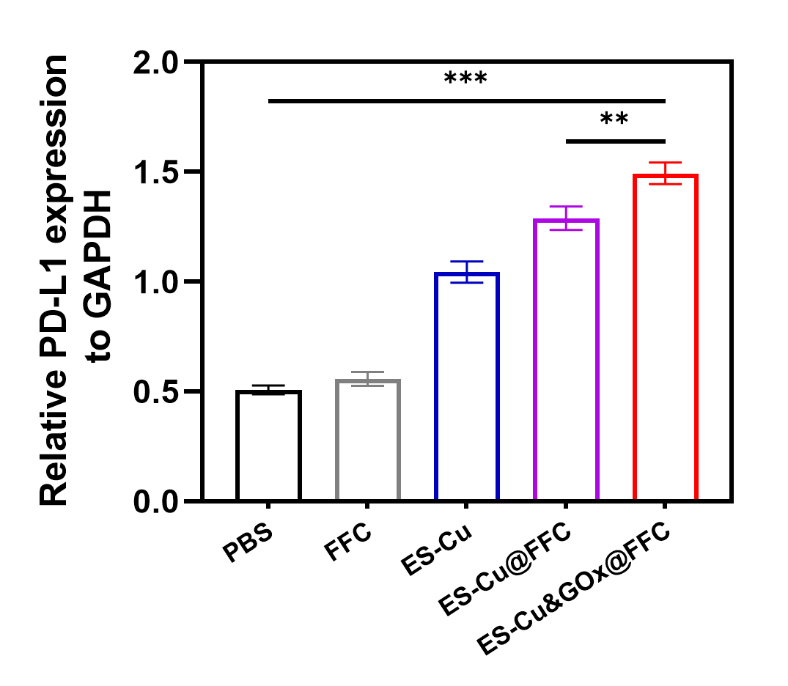
**

**Figure S12.** Quantitative analysis of PD-L1 protein expression in 4T1 cells of different treatment groups after normalization with GAPDH. The indicated results represent the mean ± SD of three independent experiments. ns, not significant, *P < 0.05, **P < 0.01, ***P < 0.001.

**
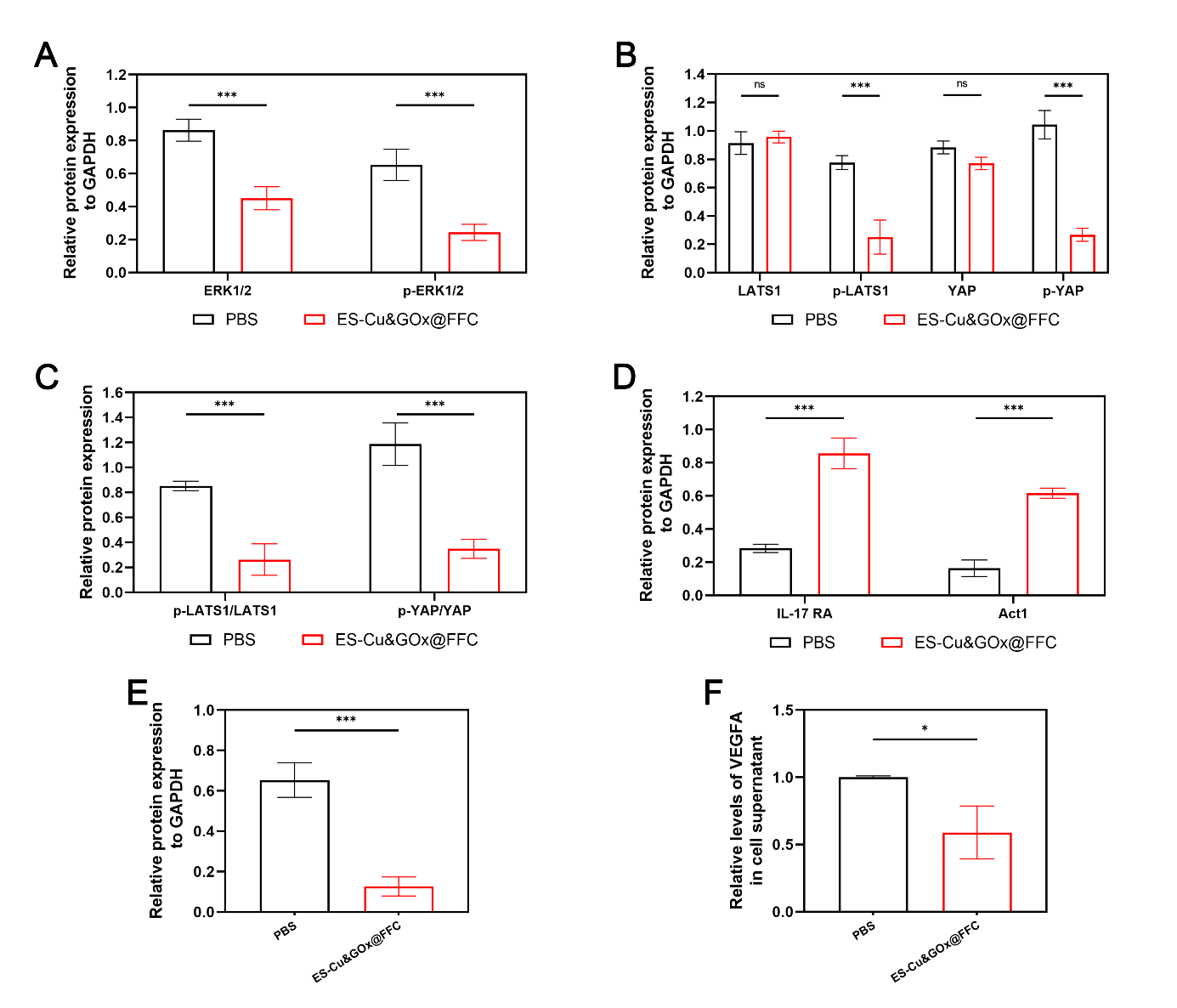
**

**Figure S13.** (A) Quantitative analysis of MAPK signaling pathway-related proteins expression in 4T1 cells of different treatment groups after normalization with GAPDH. (B) Quantitative analysis of Hippo signaling pathway-related proteins expression in 4T1 cells of different treatment groups after normalization with GAPDH. (C) The ratio of phosphorylated protein to total protein expression levels of Hippo signaling pathway-related proteins in different treatment groups. (D) Quantitative analysis of IL-17 signaling pathway-related proteins expression in 4T1 cells of different treatment groups after normalization with GAPDH. (E) Quantitative analysis of VEGF protein expression in 4T1 cells of different treatment groups after normalization with GAPDH. (F) Relative expression levels of VEGF in cell supernatants of different treatment groups. The indicated results represent the mean ± SD of three independent experiments. ns, not significant, *P < 0.05, **P < 0.01, ***P < 0.001.

**
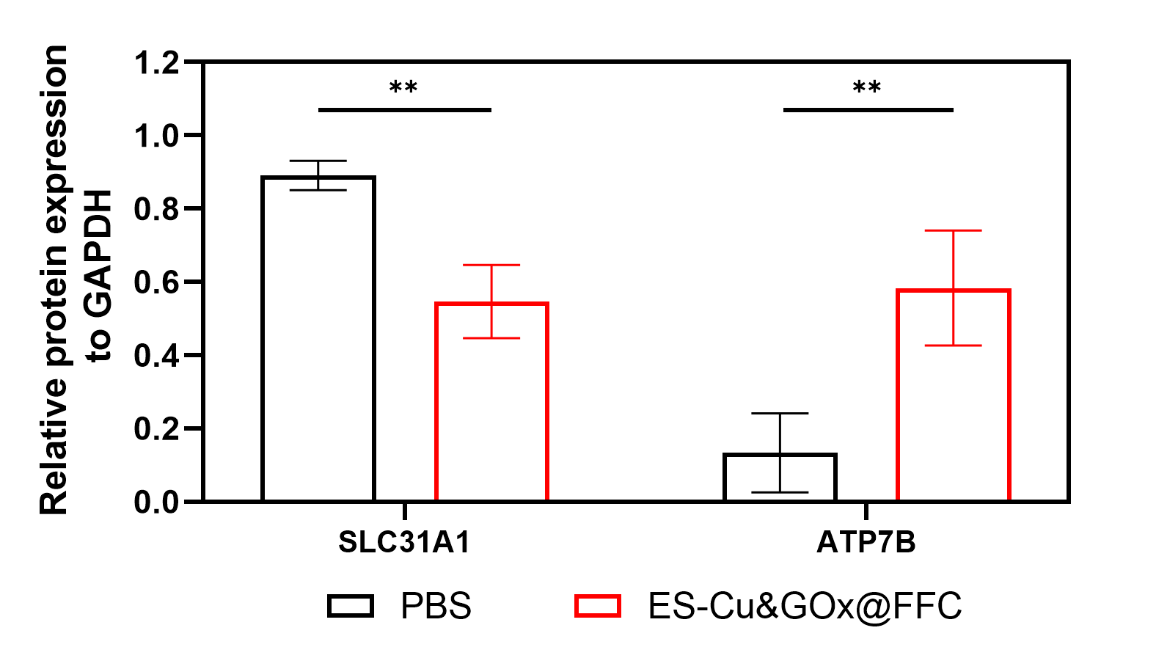
**

**Figure S14.** Quantitative analysis of copper homeostasis-related proteins expression in 4T1 cells of different treatment groups after normalization with GAPDH. The indicated results represent the mean ± SD of three independent experiments. ns, not significant, *P < 0.05, **P < 0.01, ***P < 0.001.

**
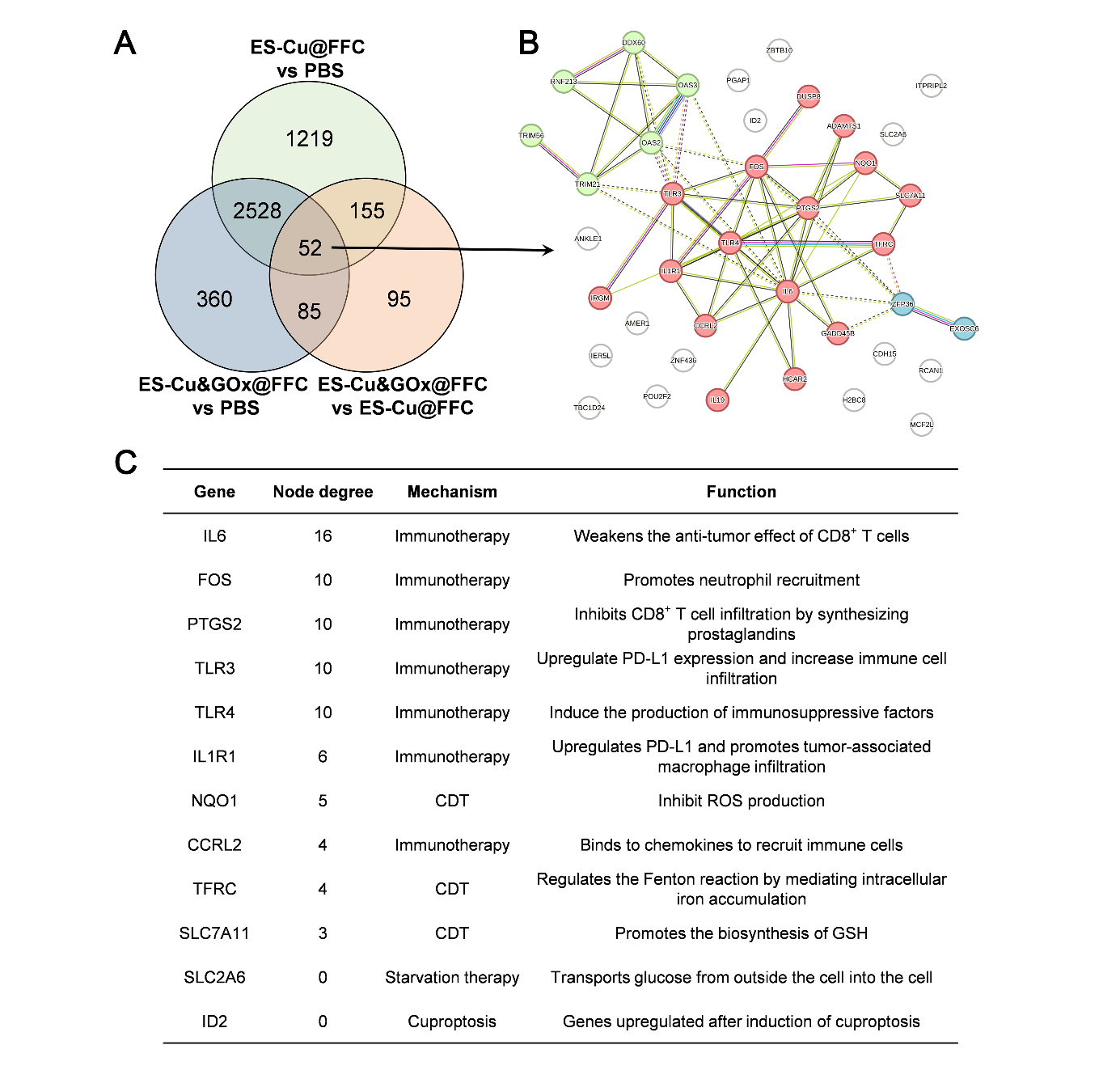
**

**Figure S15.** (A) The Venn diagram shows the number of differentially expressed genes in each treatment group. (B) PPI network of Venn diagram intersection genes. (C) The functions of hub genes and popular genes.

**
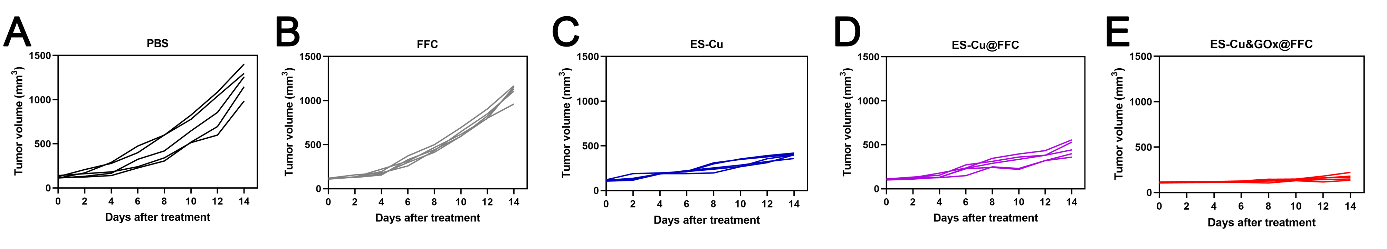
**

**Figure S16.** Change curves of single tumor volume in different treatment groups during treatment.

**
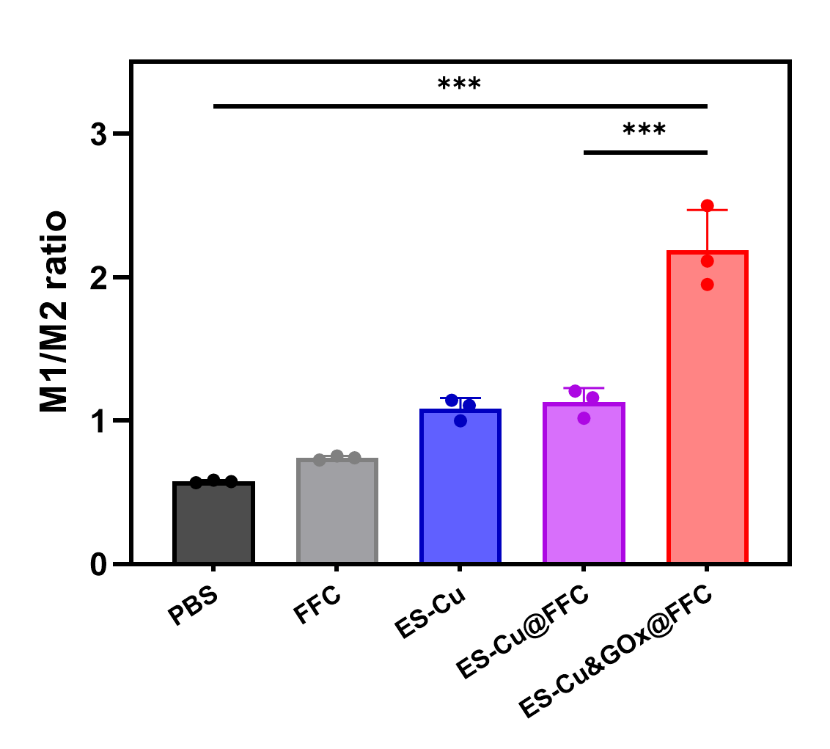
**

**Figure S17.** Quantitative analysis of M1/M2 macrophage ratio in tumor tissues of mice after different treatments. The indicated results represent the mean ± SD of three independent experiments. ns, not significant, *P < 0.05, **P < 0.01, ***P < 0.001.

**
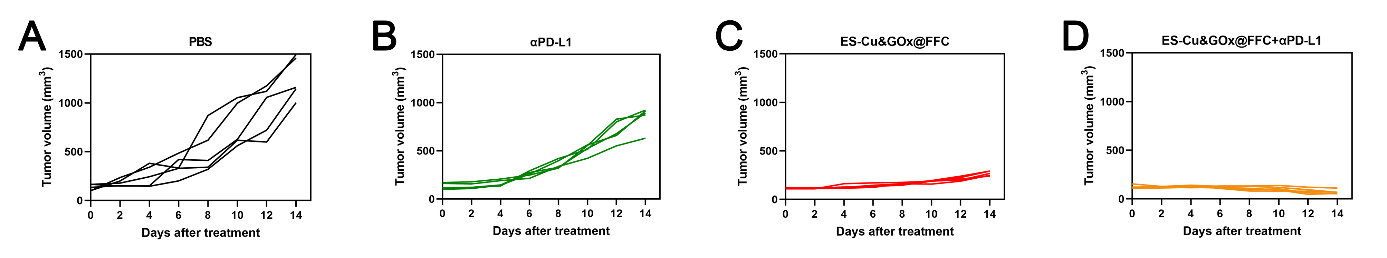
**

**Figure S18.** Change curves of single tumor volume in different treatment groups during treatment.

**
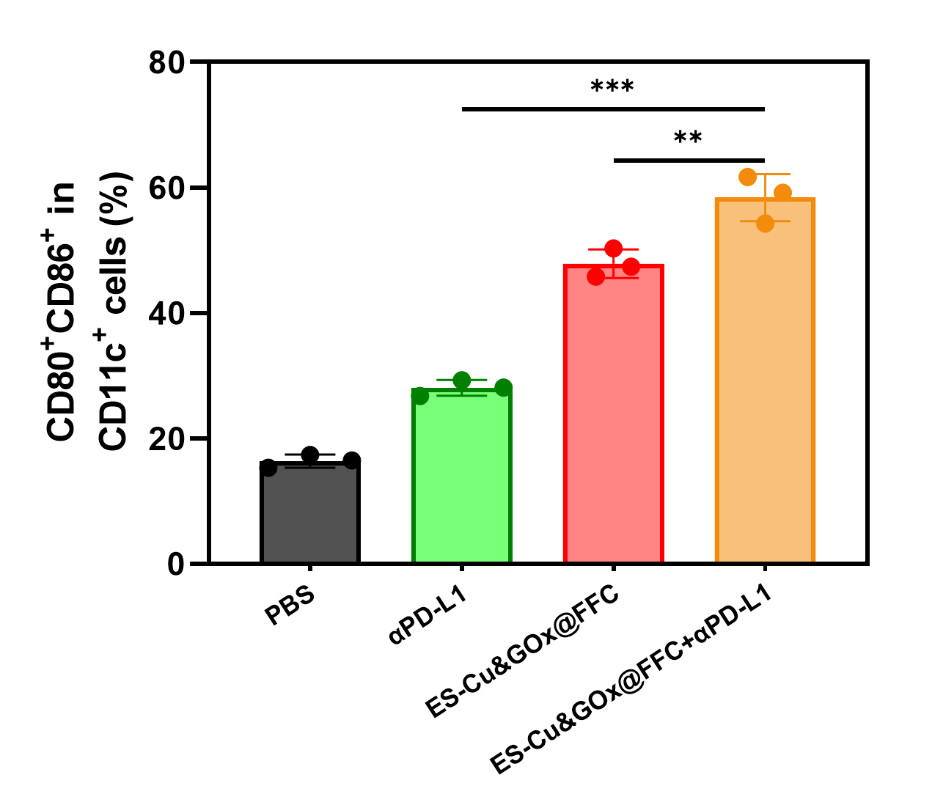
**

**Figure S19.** Quantitative analysis of DCs in tumor tissues of mice after different treatments. The indicated results represent the mean ± SD of three independent experiments. ns, not significant, *P < 0.05, **P < 0.01, ***P < 0.001.

**
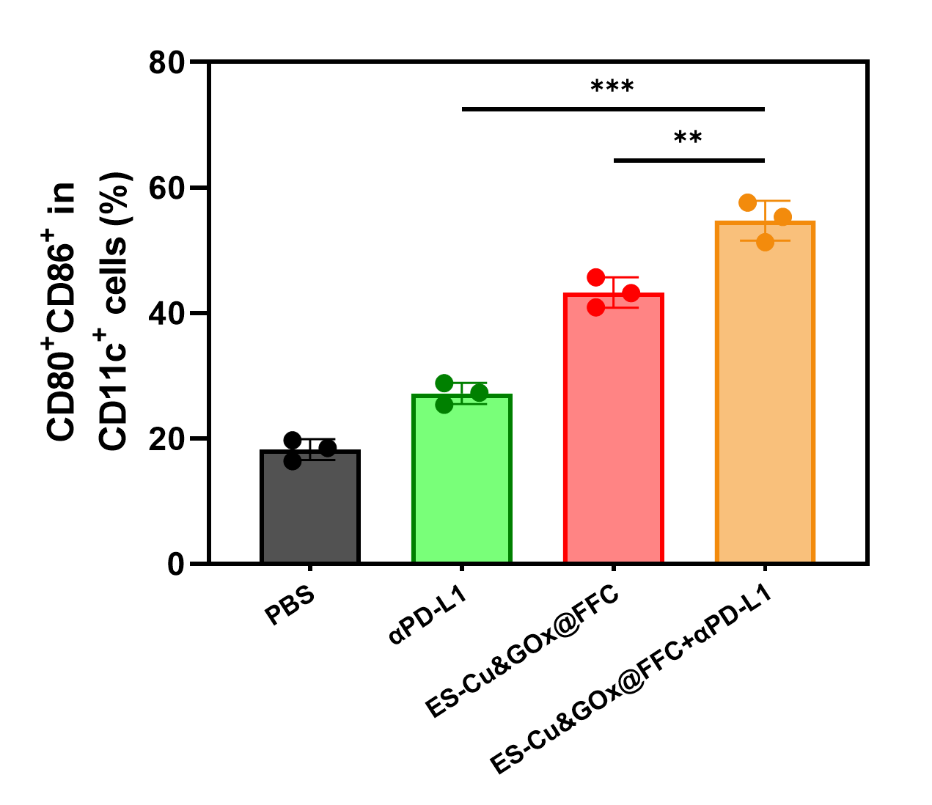
**

**Figure S20.** Quantitative analysis of DCs in the TDLNs tissues of mice after different treatments. The indicated results represent the mean ± SD of three independent experiments. ns, not significant, *P < 0.05, **P < 0.01, ***P < 0.001.

**
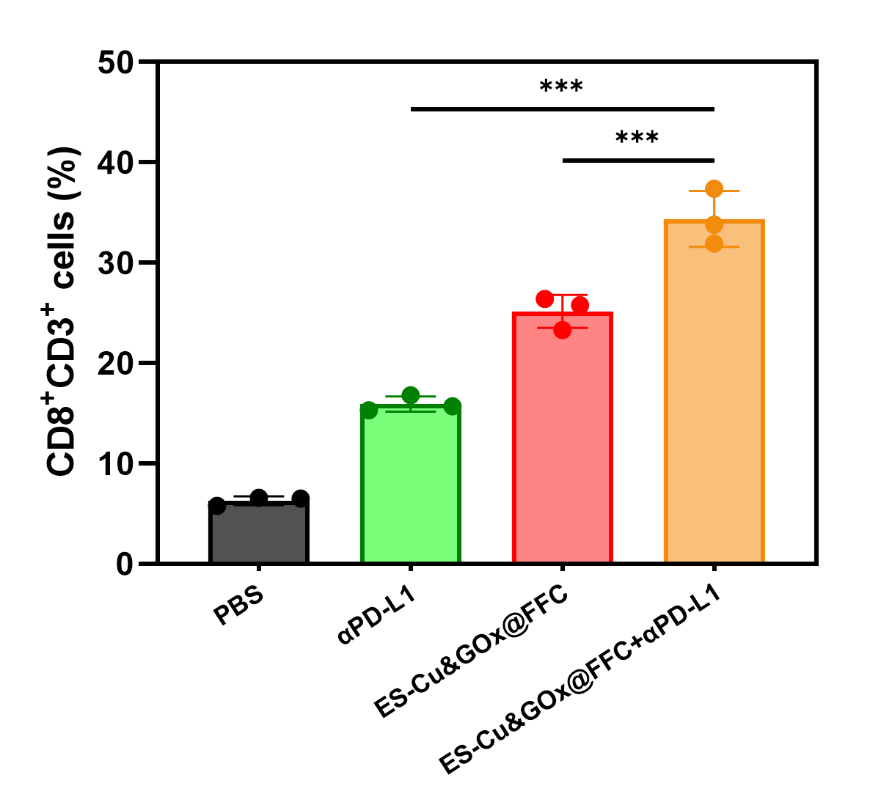
**

**Figure S21.** Quantitative analysis of CD8^+^T cells in tumor tissues of mice after different treatments. The indicated results represent the mean ± SD of three independent experiments. ns, not significant, *P < 0.05, **P < 0.01, ***P < 0.001.

**
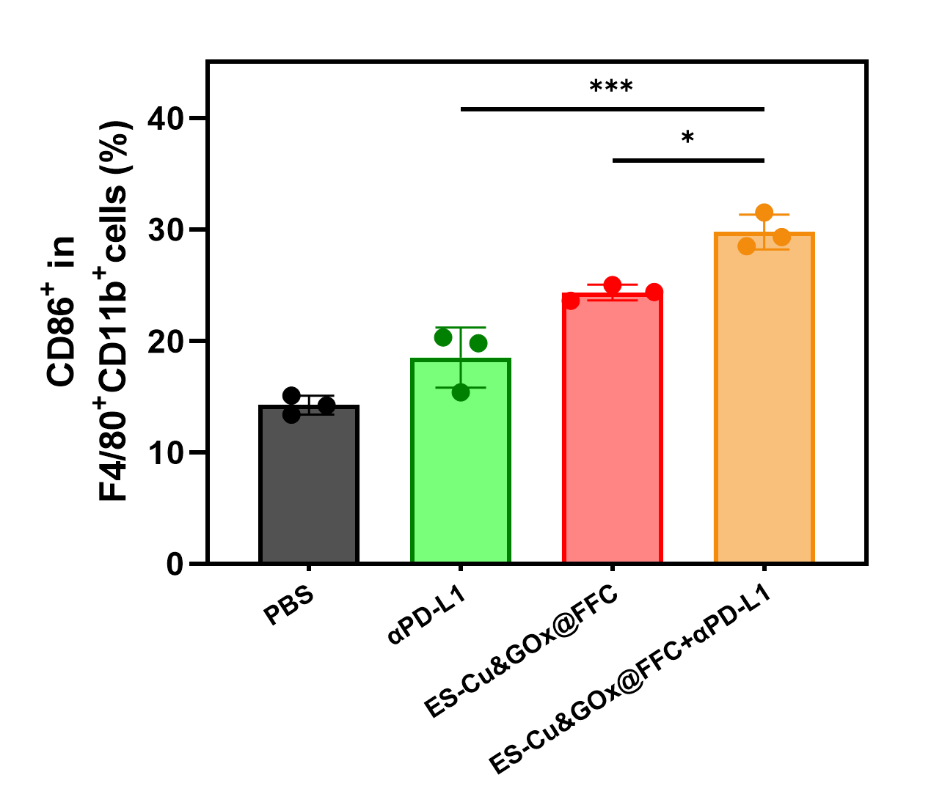
**

**Figure S22.** Quantitative analysis of M1 macrophages in tumor tissues of mice after different treatments. The indicated results represent the mean ± SD of three independent experiments. ns, not significant, *P < 0.05, **P < 0.01, ***P < 0.001.

**
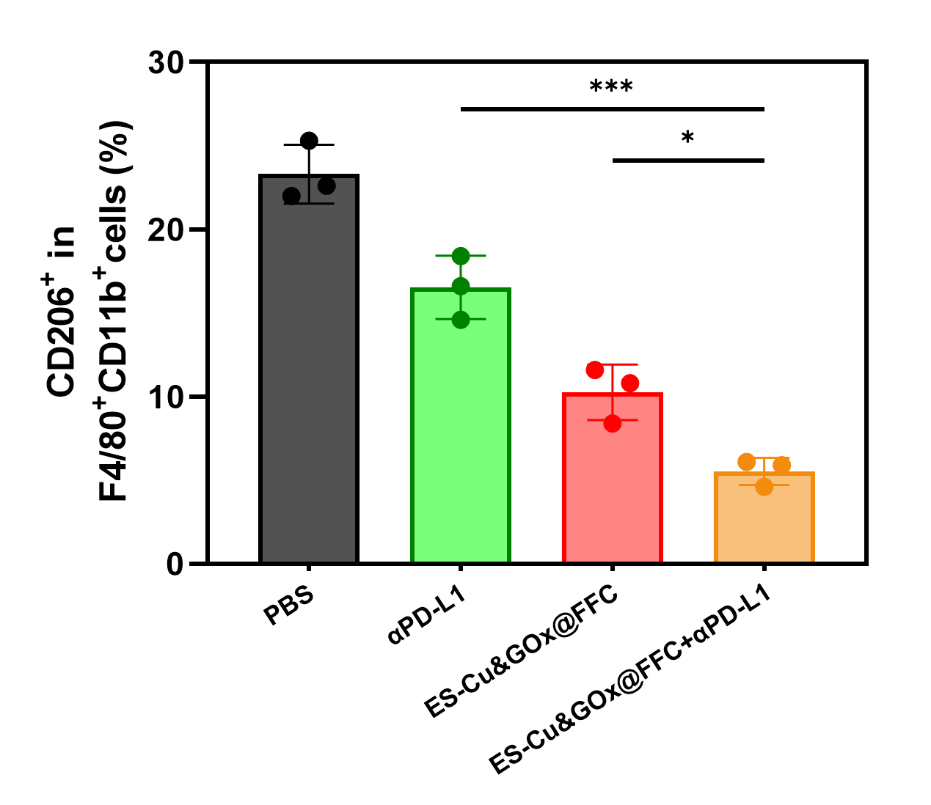
**

**Figure S23.** Quantitative analysis of M2 macrophages in tumor tissues of mice after different treatments. The indicated results represent the mean ± SD of three independent experiments. ns, not significant, *P < 0.05, **P < 0.01, ***P < 0.001.

**
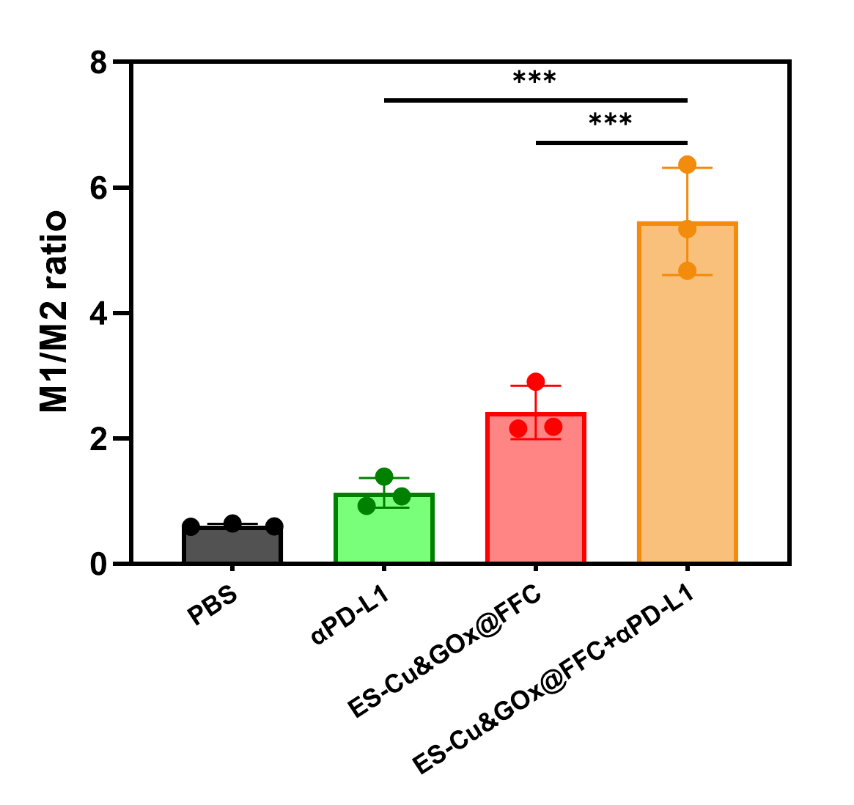
**

**Figure S24.** Quantitative analysis of the ratio of M1/M2 macrophages in tumor tissues of mice after different treatments. The indicated results represent the mean ± SD of three independent experiments. ns, not significant, *P < 0.05, **P < 0.01, ***P < 0.001.

**
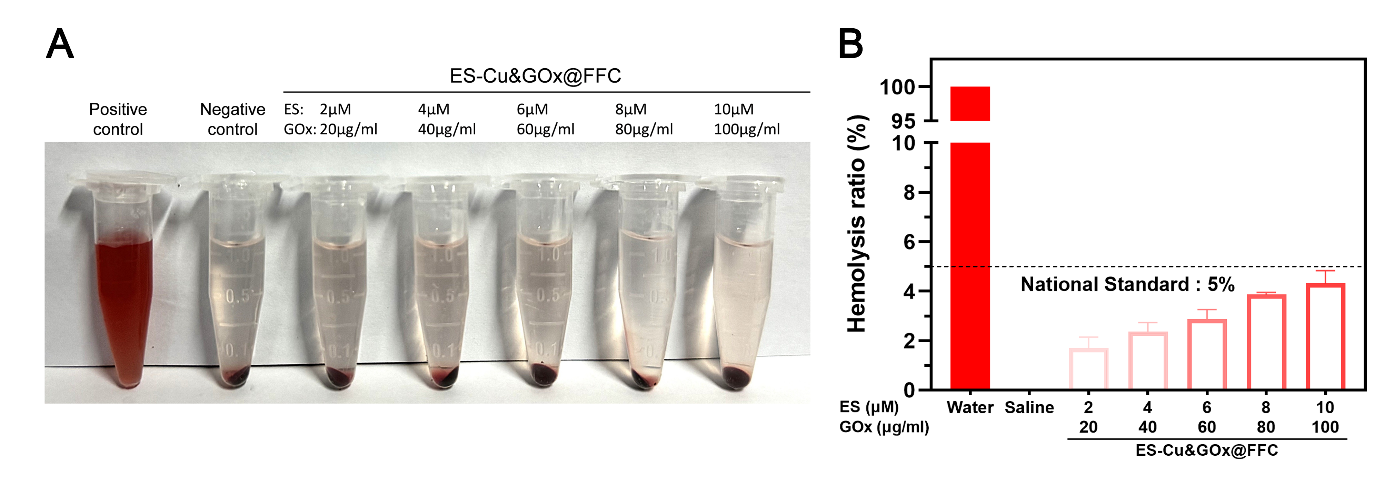
**

**Figure S25.** (A) Hemolytic status of RBCs treated with deionized water, normal saline, and ES-Cu&GOx@FFC gels of different concentrations for 1 h. (B) Statistical graph of the hemolysis rate of each experimental group. The indicated results represent the mean ± SD of three independent experiments.

**
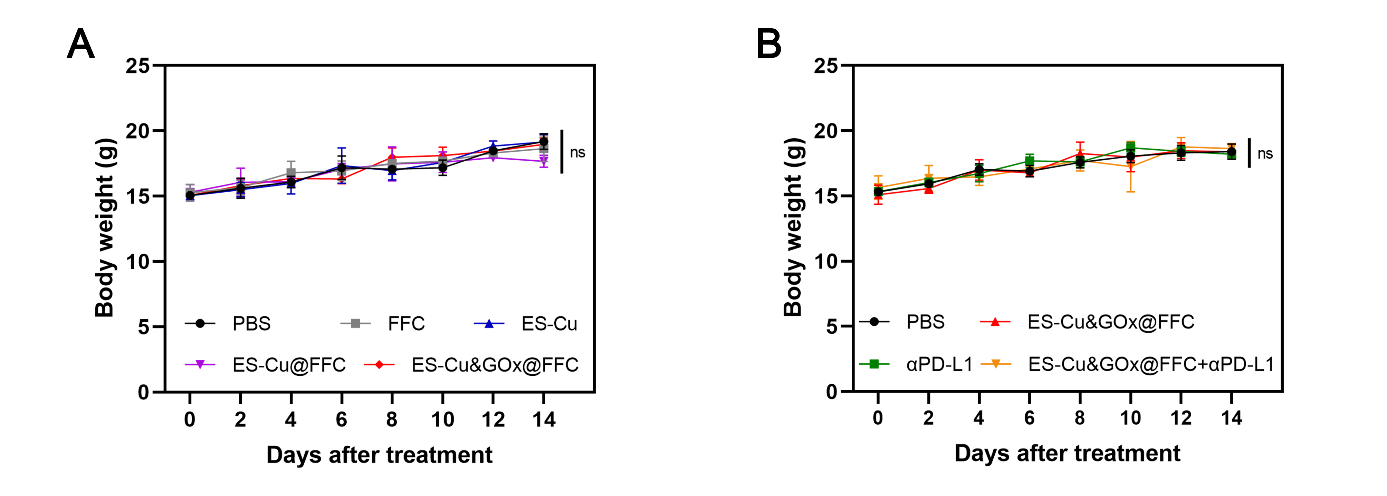
**

**Figure S26.** (A, B) The trend of body weight changes in 4T1 tumor-bearing mice after different treatments. The indicated results represent the mean ± SD of five independent experiments. ns, not significant, *P < 0.05, **P < 0.01, ***P < 0.001.

**
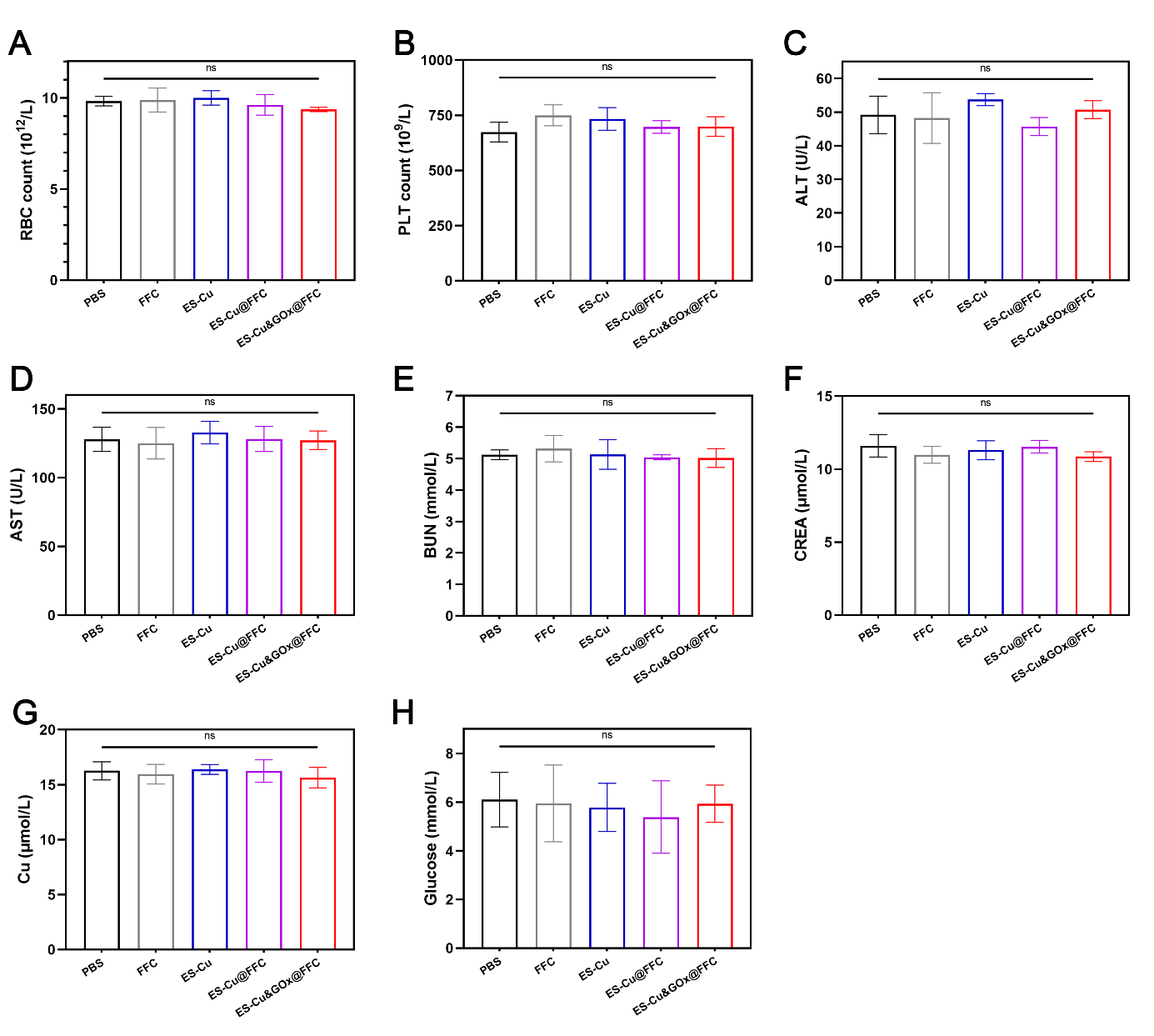
**

**Figure S27.** Blood routine and biochemical indicators of mice in different treatment groups, including RBC (A), PLT (B), ALT (C), AST (D), BUN (E), CREA (F), Cu ions (G), and Glucose (H). The indicated results represent the mean ± SD of three independent experiments. ns, not significant, *P < 0.05, **P < 0.01, ***P < 0.001.


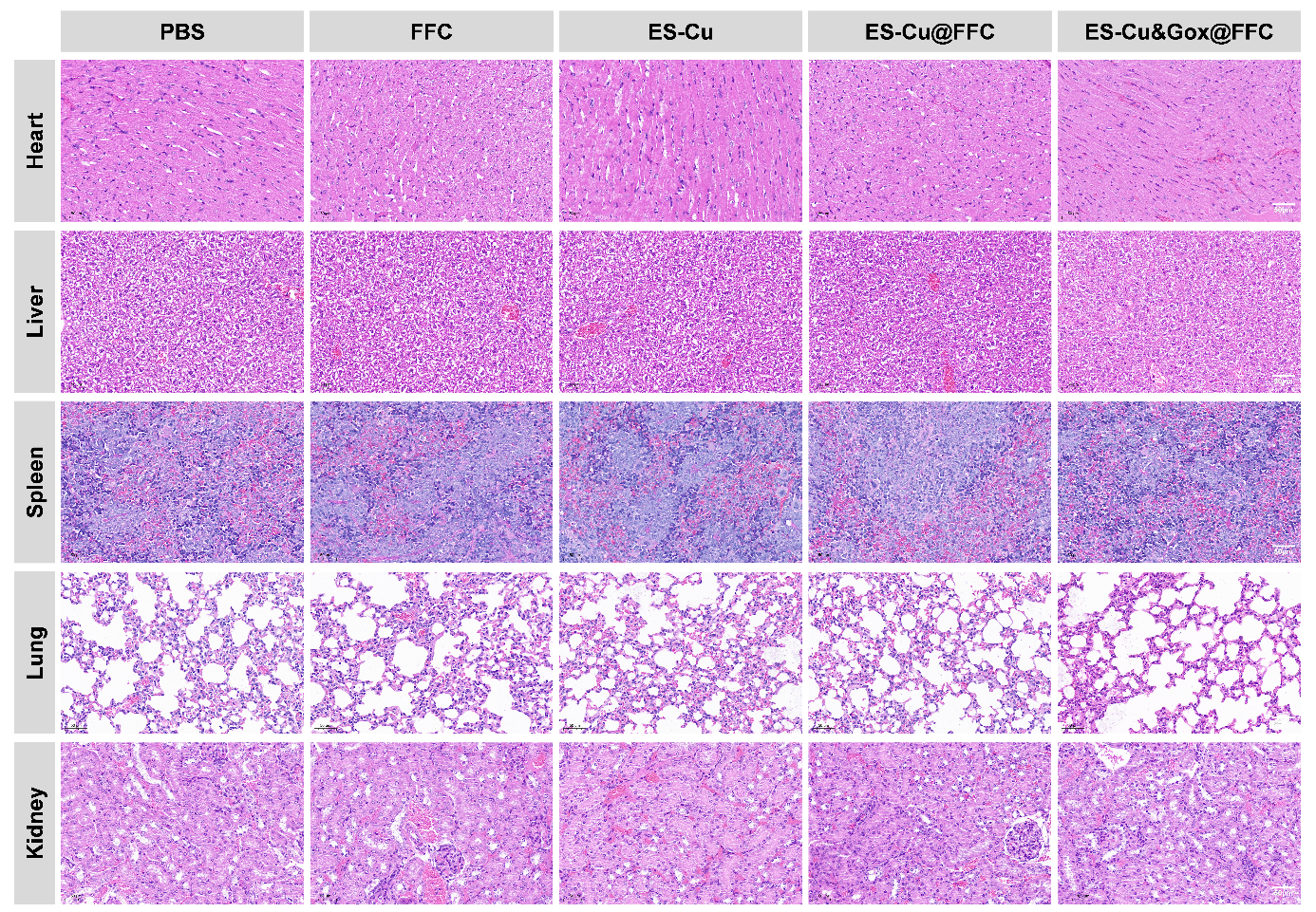


**Figure S28.** H&E staining of heart, liver, spleen, lung, and kidney tissues of mice on day 14 after receiving different treatments (Scale bar = 50μm).
